# Optimal Treatment of Tumor in Upper Human Respiratory Tract Using Microaerosols

**DOI:** 10.1021/acsomega.4c02324

**Published:** 2024-05-27

**Authors:** Hafiz Hamza Riaz, Adnan Munir, Umar Farooq, Attique Arshad, Tzu-Chi Chan, Ming Zhao, Niaz Bahadur Khan, Mohammad S. Islam

**Affiliations:** † School of Mechanical and Manufacturing Engineering, National University of Sciences and Technology, H-12, Islamabad, Pakistan; ‡ Department of Mechanical and Computer-Aided Engineering, National Formosa University, Yunlin 632, Taiwan, Republic of China; § School of Engineering, Design and Built Environment, Western Sydney University, Penrith, New South Wales 2751, Australia; ∥ Mechanical Engineering Department, College of Engineering, University of Bahrain, Isa Town 32038, Bahrain; ⊥ School of Mechanical and Mechatronic Engineering, University of Technology Sydney, Ultimo, New South Wales 2007, Australia

## Abstract

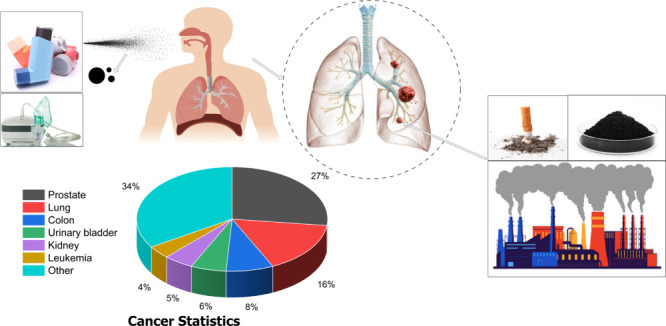

Lung cancer is a frequently diagnosed respiratory disease caused by particulate matter in the environment, especially among older individuals. For its effective treatment, a promising approach involves administering drug particles through the inhalation route. Multiple studies have investigated the flow behavior of inhaled particles in the respiratory airways of healthy patients. However, the existing literature lacks studies on the precise understanding of the transportation and deposition (TD) of inhaled particles through age-specific, unhealthy respiratory tracts containing a tumor, which can potentially optimize lung cancer treatment. This study aims to investigate the TD of inhaled drug particles within a tumorous, age-specific human respiratory tract. The computational model reports that drug particles within the size range of 5–10 μm are inclined to deposit more on the tumor located in the upper airways of a 70-year-old lung. Conversely, for individuals aged 50 and 60 years, an optimal particle size range for achieving the highest degree of particle deposition onto upper airway tumor falls within the 11–20 μm range. Flow disturbances are found to be at a maximum in the airway downstream of the tumor. Additionally, the impact of varying inhalation flow rates on particle TD is examined. The obtained patterns of airflow distribution and deposition efficiency on the tumor wall for different ages and tumor locations in the upper tracheobronchial airways would be beneficial for developing an efficient and targeted drug delivery system.

## 1 Introduction

**Figure 1:**
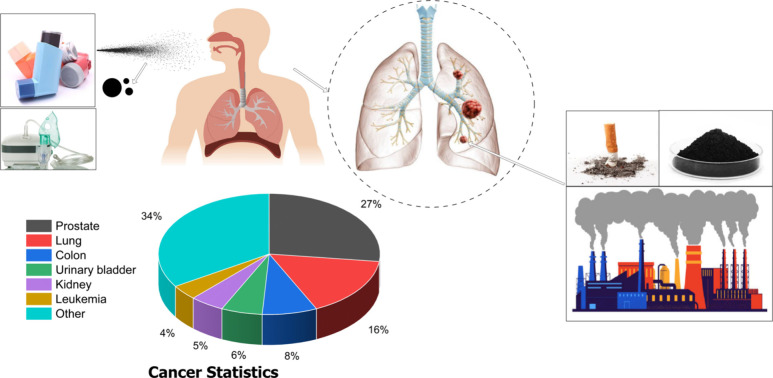
A general depiction of the application of drug inhalation devices (DPIs and nebulizers) for the treatment of lung cancer, cancer statistics, and factors contributing toward lung cancer.

Environmental exposure, mutations, and particulate matter in polluted air are factors associated with lung cancer.^1−3^ Cigarette smoke (active and passive) is the leading cause of lung cancer and is responsible for around 90% of lung cancer deaths.^4^ These environmental factors lead to glomus tumors, which are rare growths that come from glomus cells and look a bit like a smooth muscle.^5^ The unrestricted growth of these cells in the respiratory regions makes up a lump of mass which is termed a lung tumor or cancer. Lung cancer was the leading cause of cancer death by 2020, and currently, it is the second most diagnosed cancer with an estimated 2.2 million new cases and 1.8 million deaths. It contributes around 11.4% of the total cancers diagnosed and is responsible for 1 in 5 (18%) cancer-related deaths.^6^ The presence of a tumor in the respiratory airways partially blocks the airflow path and affects the breathing capacity of a person. Drug aerosol intake in the human respiratory tract through inhaling devices like dry powder inhalers (DPIs) or nebulizers is an effective means of treating lung diseases and other respiratory illnesses.^3,7^ Moreover, inhaling medicinal drugs has a negligible risk of adverse side effects which are associated with traditional drug delivery methods.^8^ Hence, adequate deposition of medicinal aerosols to the targeted areas of unhealthy respiratory airways like a tumor surface or swollen region is a convenient way for prompt healing. Figure 1 provides a comprehensive illustration of lung tumors, commonly used inhalation devices for treating tumor cells, the global incidence of lung cancer relative to other types of cancer, and the environmental factors that contribute to their development.

Engineers and scientists have employed computational fluid dynamics (CFD) to study the airflow, transportation, and deposition (TD) of medicinal aerosols and particulate matter in polluted air through the complex pathways of the human respiratory system.^9−22^ The number of numerical studies conducted on unhealthy lungs in comparison to healthy lungs is less in the existing literature. CFD can assist researchers in understanding the flow physics of unhealthy lungs and how aerosols move within them for better treatment of pulmonary diseases. Segal et al.^23^ performed numerical simulations to study the growth of the side wall tumor on the carinal ridge in the respiratory airways of a 4-year-old patient. They found that the carinal ridge tumor had a significant effect on the flow patterns and the flow downstream of the tumor. Based on the evaluation of respiratory flow, Yang et al.^24^ reported that the presence of any obstructive medium in the airways has direct effects on the airflow velocity streamlines. They also performed a detailed comparison of the airflow behavior and wall shear stress under various ventilatory settings and in both healthy and unhealthy lungs. Sul et al.^25^ generated lung models with no obstructions and with symmetric and random obstructions. They demonstrated a significant difference between the flow patterns in healthy and unhealthy lungs, more so during the expiratory flow as compared to the inhalation phase. All these studies focused on the variations of airflow patterns, but only a few have investigated the behavior of drug deposition in an obstructed or unhealthy airway tract. Martonen and Guan^26^ investigated the effects of the size of lung tumors on the airflow patterns and the effect of particle size on the particle TD in the respiratory pathways. Particles of three different sizes (0.21, 3.50, 7.24 μm) were injected, and it was found that small particles were deposited less on the tumor surface compared to larger particles due to a weak impact mechanism. Additionally, when the tumor size ratio (*r*/*R*) was varied from 0 to 2, it was observed that localized flow patterns were dominant for tumors with a size ratio of 0.8 or less.^27^ Kleinstreuer and Zhang^3^ numerically investigated the existence of single and double hemispherical side wall tumors in the fifth generation of an ideal respiratory tract model. They found that the tumor size and location have substantial influence on the deposition fraction of inhaled drugs. Around 11% of particles deposited on the tumor surface for a critical tumor radius *r*/*R* = 1.25 with a constant inlet flow rate of 60 L/min. Luo et al.^28^ investigated the behavior of particle deposition in an unhealthy lung suffering from a chronic obstructive pulmonary disease which causes inflammation and narrowing of airways just like a tumor. Srivastav et al.^29^ studied the influence of a polypoid tumor on the behavior of wall shear stress and deposition of inhaled particles in the lower region of the trachea. They reported high flow disturbances near the tumor region. Similar findings were reported by Singh^30^ in a study on the effect of the presence of a glomus tumor in the trachea on the airflow streamlines, secondary flows, and particle deposition. Growth of glomus tumors in the respiratory tract can occur between the age groups of children and adults and in various locations of the respiratory tract such as the trachea, major lobes, and main airway branches.^31−34^ Studying the impact of tumors on airflow and particle deposition in bronchial airways can be beneficial for health assessment studies, as well as for analyzing drug-aerosol delivery. Singh^30^ and Srivastav et al.^29^ performed simulations on unhealthy respiratory models which included glomus tumors in the trachea region of the lung. Kleinstreuer and Zhang^3^ studied the effect of the location and size of a hemispherical tumor in the fifth generation of the lung model on the particle deposition on tumor surfaces. However, they did not study the age-specific particle deposition as well as the effect of a wide range of particle diameters on the TD efficiency in the upper airways of the unhealthy lung. The global incidence of lung cancer is increasing, with diagnosis often occurring around the age of 70.^35^ Hence, it is essential to have a clear understanding of drug particle deposition in the respiratory tracts of aged individuals, to facilitate optimized treatment of the disease.

The objective of this study is to numerically investigate the impact of a glomus hemispherical tumor in the upper airways of the age-specific lungs on the transport and deposition of microscale particles. To achieve this objective, three-dimensional ideal lung models with symmetric and planar pathways from G3 to G6 are generated for ages 50, 60, and 70 years. The G3–G6 section is used to simulate the effects of upper airway tumors. Additionally, this approach neglects the cartilaginous rings present in the larynx, trachea (G0), and main bronchi (G1). The study analyzes in detail the airflow distribution and particle deposition on the tumor surface for each age-specific lung model and compares them using 3D numerical simulations. The effect of particle size and inlet airflow rate on deposition efficiency is also studied.

## 2 Lung Geometry

Three-dimensional lung models with symmetric and planar pathways from generation three to six are generated for ages 50, 60, and 70 years. According to the designed models used by Xu and Yu,^36^ the geometric parameters of the age-specific lung models used in this study are listed in Table 1. The variations in the geometrical parameters for an adult lung are negligible from the age of 30 to 50.^37^ This led to the assumption that a 30-year-old lung falls in the same group as a 50-year-old lung in terms of geometrical parameters. From 50 years old onward, the airway diameters for each generation shrink by 10% after every 10 years,^38^ and the tissues of the lung become 7% hardened especially between the ages of 50 and 80.^39^

**Table 1:** Geometric Parameters of Lung Model Used by Xu and Yu^36^ (Adapted with Permission from ref (36). Copyright 1986 Taylor & Francis)

	Airway Diameter (mm)			
Generation Number	50 year	60 year	70 year	Airway Length (mm)
3	5.60	5.04	4.48	7.59
4	4.50	4.05	3.60	12.68
5	3.50	3.15	2.80	10.71
6	2.80	2.52	2.24	9.01

**Figure 2:**
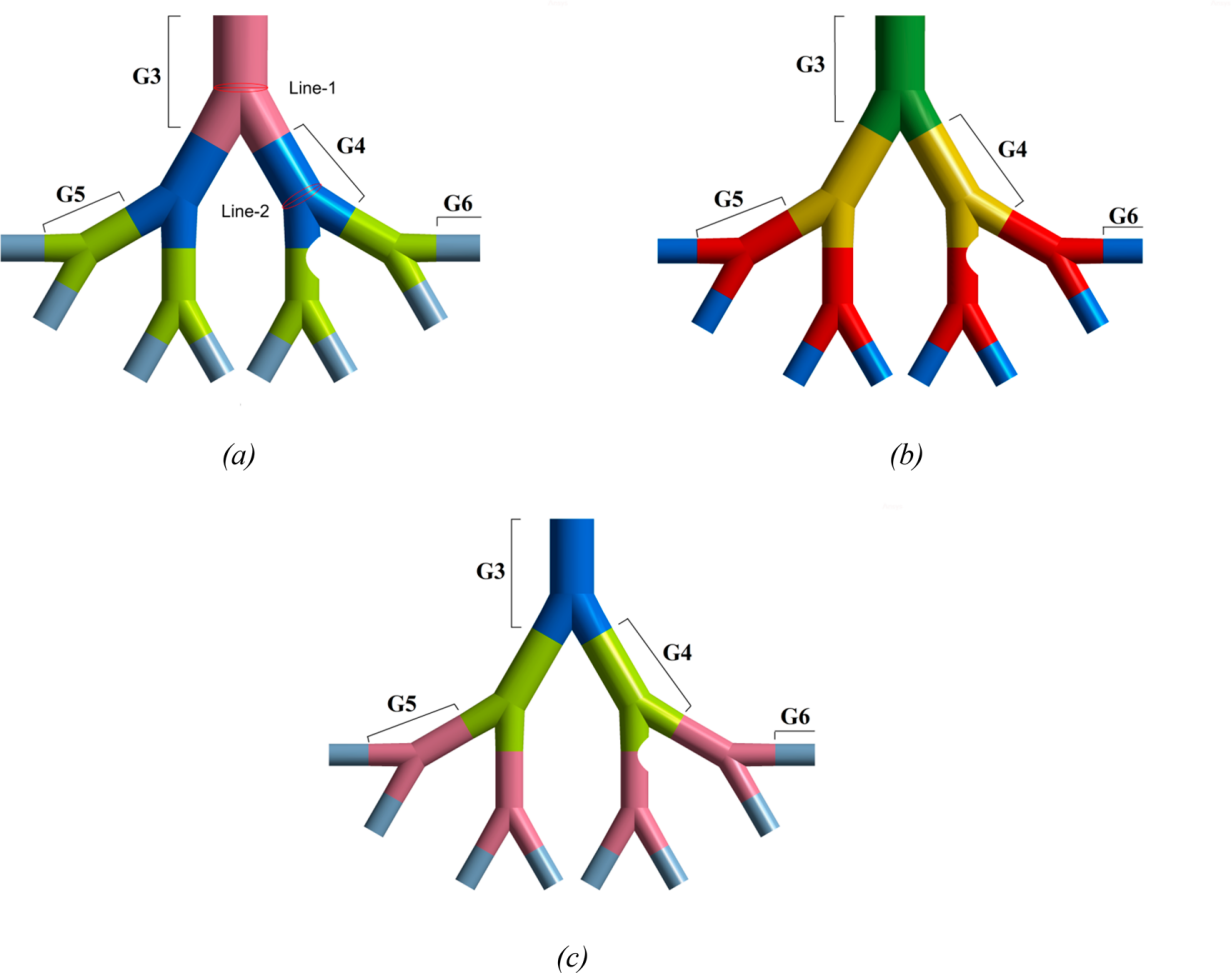
Tracheobronchial lung airway models (G3–G6) with sidewall tumors for the 50- (a), 60- (b), and 70- (c) year-old lung.

Figure 2 shows the lung models with a tumor in the fifth generation for each age group. The airway length for each generation remains constant, but its diameter reduces with the increase of age. Only four generations are simulated because studying the complete lung model demands unaffordable computational time. An effective cutting method is employed to create a sidewall hemispherical glomus tumor in G5 of each lung model. The location selected for the sidewall tumor corresponds to the published data available in the literature.^3,23,40^ Moreover, the location of the tumor is also appropriate in the sense that it does not impact the aerosol distribution and profiles of the inlet air velocity in the third generation. The blockage of the tumor to the airflow in the airway can increase from 20% at the mild stage to 80% at the late stage.^41^ The radius (*r*) of the hemispherical tumor on the airway wall is selected based on the radius of the local pathway (*R*) in which the tumor is present and is defined by the ratio *r*/*R*. Using the geometrical parameters listed in Table 1 and choosing the ratio for the tumor size as *r*/*R* = 1.25, which corresponds to around 50% blockage of the airway,^3^ the lung models for each age are modeled on SolidWorks and are demonstrated in Figure 2.

## 3 Numerical Method

The simulations are performed using the ANSYS Fluent 19.0 software. For the airflow in airways, the governing equations are the Reynolds-averaged Navier–Stokes (RANS) equations:
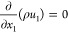
1
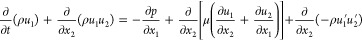
2where ρ is the density of the air, which is 1.225 kg/m^3^, μ is the dynamic viscosity, taken as 1.79 × 10^–5^ kg/ms, *p* is the pressure of air, *u*_1_ and *u*_2_ are the fluid velocity in the horizontal and vertical directions, respectively, and *x*_1_ and *x*_2_ represent the horizontal and vertical directions in the fluid, respectively. The Shear-Stress Transport (SST) *k*–ω turbulence model is used to simulate the turbulence, as it is proved to be more suitable for the adverse pressure gradients.^42^ The SST *k*–ω model is a modification of the standard *k*–ω model to efficiently incorporate the robust and correct formulation in the near-wall sections with the free-stream independence in the far-field section.

The SST *k*–ω turbulence model integrated with the essential blending functions is given in the following equations:
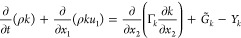
3
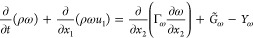
4Γ_*k*_ and Γ_ω_ represent the effective diffusivity of turbulent kinetic energy (*k*) and dissipation rate (ω), respectively, in eqs 3 and 4. *G̃*_*k*_ represents the generation of turbulence kinetic energy (*k*), and *G̃*_ω_ represents the generation of a specific dissipation rate (ω). *Y*_*k*_ and *Y*_ω_ represent the dissipation of turbulent kinetic energy and dissipation rate, respectively. This model has proved to generate satisfactory results, resolving the high-pressure gradient flows near the wall and predicting accurate outputs at the boundary layer.^43−46^

For the interpolation of the diffusion and pressure gradients, the least-squares cell-based technique is employed. A pressure–velocity coupling scheme along with the second-order upwind scheme for the turbulent kinetic energy, specific dissipation rate, and momentum is employed. Moreover, a second-order implicit scheme is used to solve the numerical equations. A constant inlet velocity and zero-gauged pressure outlet boundary conditions have been applied for each lung model.^47,48^ The required inlet air velocity during the inhalation phase is calculated based on the diameter of the inlet surface of G3. For the turbulence, the specification method used is intensity and viscosity ratio with values of turbulent intensity and turbulent viscosity ratio of 5% and 10, respectively. A constant velocity is used instead of an unsteady velocity inlet profile to examine the impact of the aging effect on the deposition of particles on the tumor without the influence of variations in the velocity. The wall of the lung model and tumor is assumed to be stationary, and a no-slip condition is applied on the wall surfaces as well. As the airflow originates from either the nasal or oral cavity, passes through the trachea, and enters the airways, it is noteworthy that the maximum calculated Reynolds number at the trachea for the 50-year, 60-year, and 70-year-old lung models is 4877, 5417, and 6096, respectively. These findings lend further support to the application of a turbulence model within the scope of this study. The present model uses one-way coupling between the primary and discrete phases, which focuses only on the particle motion in airflow and ignores any kind of influence on the airflow by the particles. The choice of coupling depends upon the concentration of the secondary phase (particle) in the primary phase (air). For all of the drug delivery applications, the disperse phase concentration is well below 15%, which allows the use of one-way coupling instead of a two-way coupling model.^49^ The particle–particle interaction can be ignored in the numerical simulations as the particle suspension injected into the inlet of the lung airway model is dilute.^50^

In this study, the Discrete Phase Model (DPM) is employed for the interaction of the discrete and continuous phases. The Lagrangian approach is used to simulate the particle TD in the lung airways. The dynamics of each particle are controlled through the force balance equation:
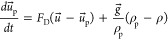
5where *u* and *u*_p_ represent the velocity of continuous and discrete phases, respectively, *g* is the gravitational acceleration, ρ_p_ is particle density, which is taken as 1100 kg/m^3^.^51−53^*F*_D_(*u⃗* – *u⃗*_p_) represents the drag force per particle mass, and the *F*_D_ for the spherical particle is determined using the following relation:
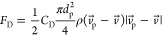
6where *C*_D_ is the drag coefficient, *d*_p_ is the diameter of the particle, and *v*_p_ is the particle velocity. For particle deposition, a trap condition is applied on the wall of the lung model and an escape condition is applied at all outlets of the airways, which corresponds to the exit point at generation six.^54,55^ This ensures that when a particle strikes the inner wall of the lung, the fate of that particle will be considered trapped or deposited at that point of the airway.

In the numerical simulations, 30 000 spherical particles with a constant particle size are injected at once from the inlet face of the lung section. The study utilizes particles with sizes ranging from 5 to 20 μm. Particles smaller than 5 μm are not considered in the simulation due to the impact of Brownian motion, which falls beyond the study’s scope. Addressing ultrafine particles necessitates a more intricate modeling approach involving distinct correction factors. The current investigation concentrates on inertial impaction and Stokes numbers, hence omitting consideration for smaller particles. A future study will focus on the dynamics of these smaller particles, exploring their behavior and characteristics. The deposition efficiency for any region is defined as the ratio of the number of particles trapped in the targeted region to the total number of particles injected at the inlet surface. Eq 7 shows the expression used for calculating deposition efficiency in this study.
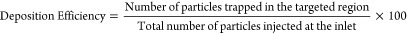
7

### 3.1 Mesh Dependency Study

**Figure 3:**
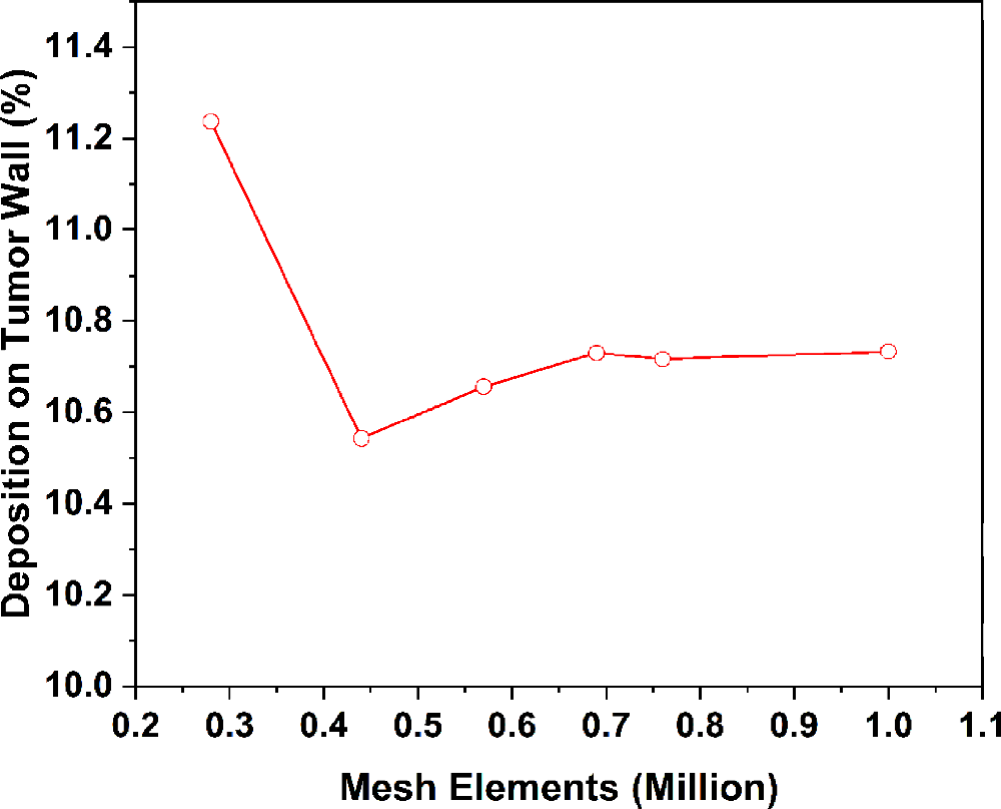
Variation of deposition efficiency at the tumor wall as a function of the number of elements of the 50-year-old lung model, with a particle diameter of 7 μm and inlet flow rate of 60 L/min.

**Figure 4:**
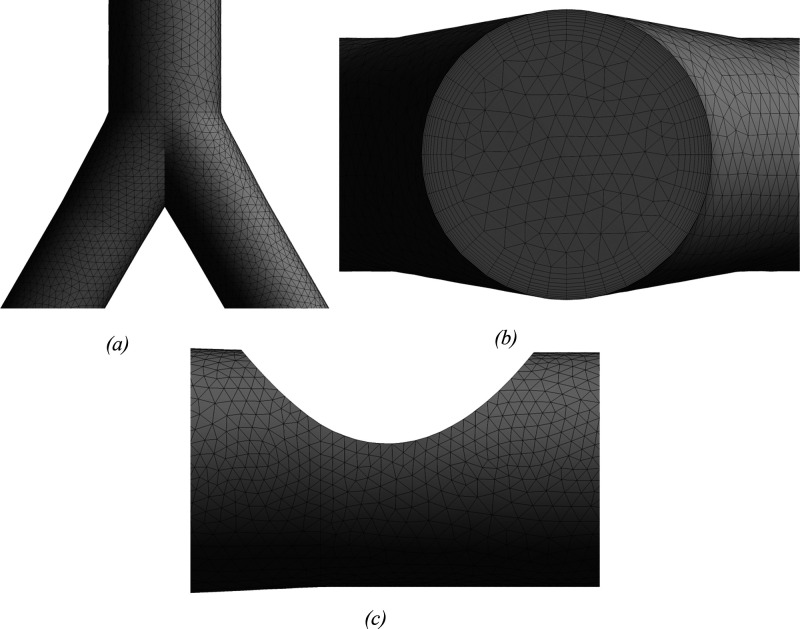
Computational mesh for the lung model. The mesh resolution on the airway wall (a). Refined inflation mesh near the inlet wall (b). Mesh density near tumor wall (c).

**Figure 5:**
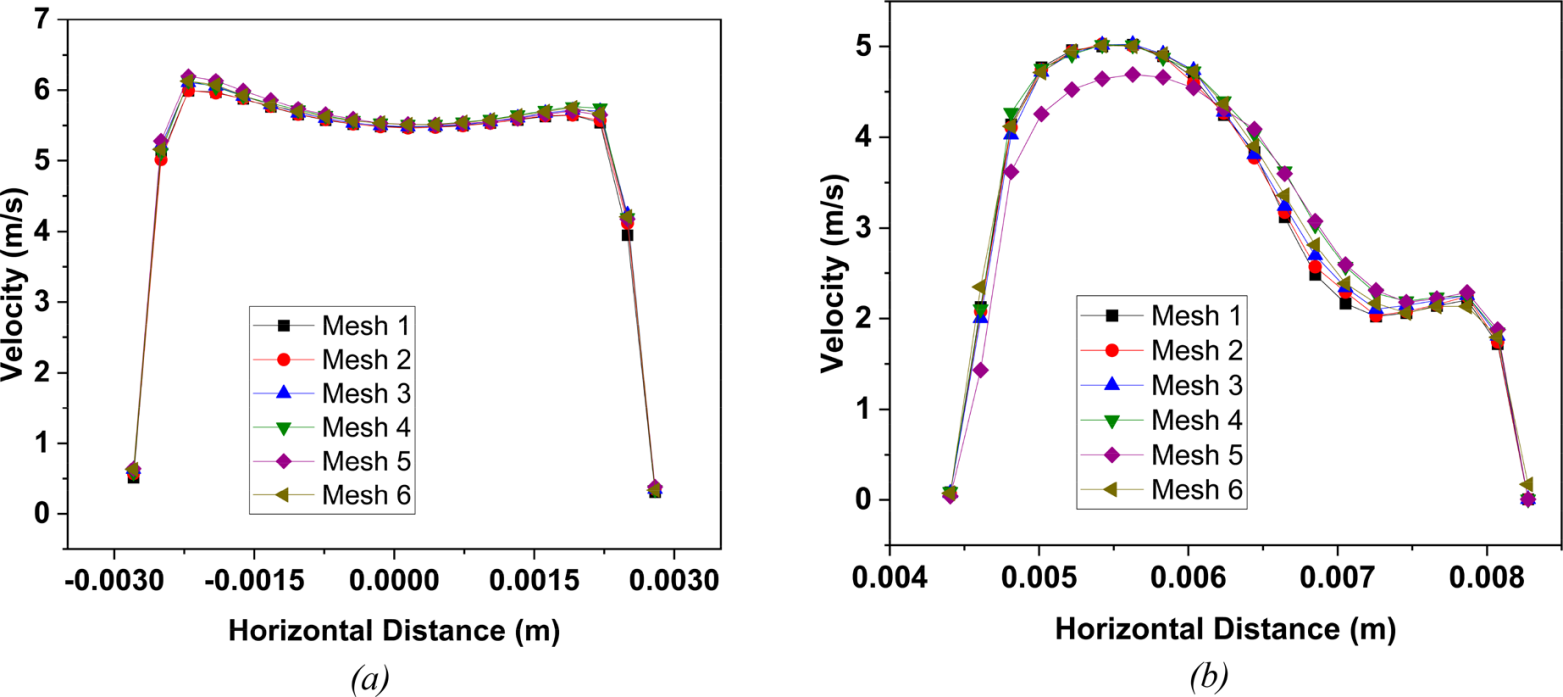
Velocity profile along (a) line 1 and (b) line 2 for six different meshes (see Figure 2 for line locations)

The mesh dependency study is carried out by performing multiple numerical simulations of the 50-year-old lung model with generations G3–G6 at an inlet flow rate of 60 L/min using the injected particle diameter as 7 μm. A total of six meshes were constructed with the same structural properties but different mesh densities. The tetrahedral element number of the meshes ranged from 286 966 in mesh 1 to 1 016 872 in mesh 6. The changes in the deposition efficiency of particles on the lung wall of G3 with a varying number of meshed elements are shown in Figure 3. The deposition efficiency maintains almost a constant value after mesh 4 with 689 804 elements. Mesh 5 with 761 052 tetrahedral elements is selected for all of the numerical simulations and analysis. Figure 4 demonstrates the mesh at the airway walls and inlet section of G3. A total of 10 layers of inflation are used near the wall for the accurate prediction of the flow of the wall surface inside the lung model. The mesh at the bifurcation of G3 and mesh density near the tumor wall is also demonstrated, and the mesh structure of each of the six meshes is the same as the one illustrated in Figure 4. Furthermore, the velocity distribution over line 1 and line 2 for all six meshes is shown in Figure 5. Clearly, the profile for each mesh follows a similar trend, and a negligible difference is found in the velocity profiles of all meshes for both lines.

### 3.2 Validation of Model

**Figure 6:**
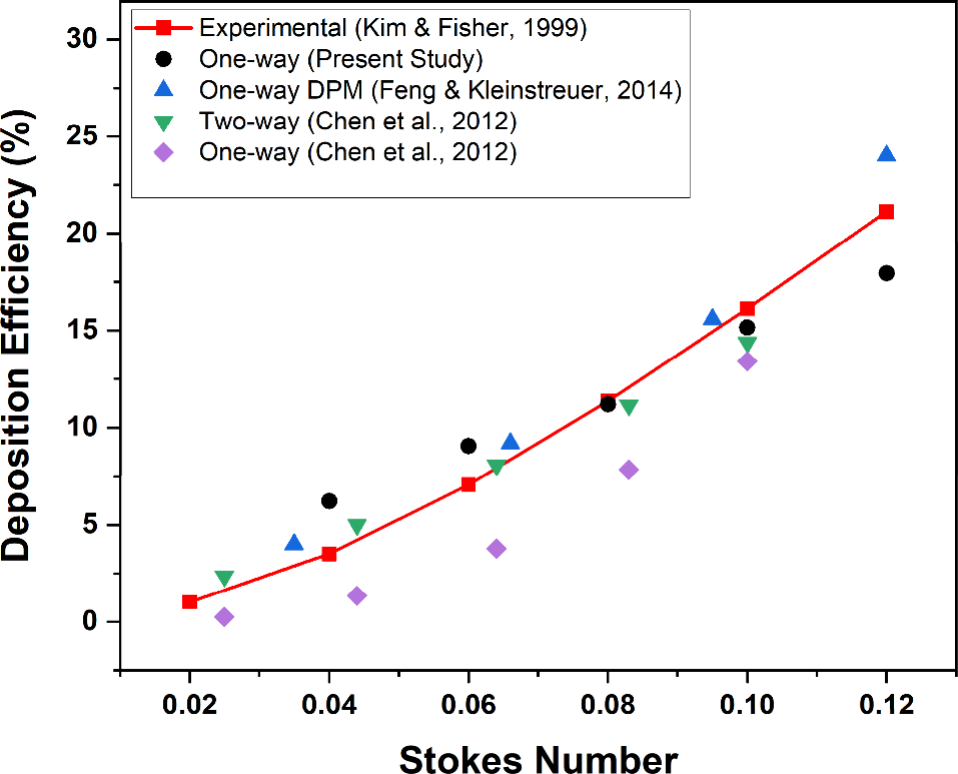
Comparison of the deposition efficiencies obtained for the first bifurcation of the G3–G6 lung model at various Stokes numbers between the present study and published experimental and numerical results.^56−58^ Reproduced with permission from ref (56). Copyright 1999, Taylor & Francis. Reproduced with permission from ref (57). Copyright 2014, Elsevier. Reproduced with permission from ref (58). Copyright 2012, Elsevier.

The numerical model used in the present study is validated against the published experimental and numerical results of particle deposition in the lung sections G3–G6.^56−58^ Various particle diameters ranging from 3 to 7 μm and a flow rate of 60 L/min for the 50-year-old healthy lung model are used for conducting simulations, and the results from the current model are found to be in good proximity with the experimental and numerical results at different Stokes numbers as demonstrated in Figure 6. Stokes number (Stk) is a dimensionless parameter used to describe the interaction between a particle and a fluid flow. It is defined as the ratio of the particle response time (the time required for the particle to respond to the fluid forces) to the characteristic time scale of the fluid flow:^59^
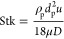
8where *D* represents the airway diameter of the inlet of G3 through which the particles are being injected and *u* is the mean inlet velocity of the air.

## 4 Results and Discussion

The simulations are performed for each lung model using an inlet flow rate of 60 L/min and multiple particle diameters ranging from 5 to 20 μm. The effect of various inhalation conditions is investigated by conducting the simulations using three inlet flow rates: 60 L/min, 45 L/min, and 30 L/min, which represent heavy, moderate, and light breathing conditions, respectively, for the 50-year-old lung model.^29^

### 4.1 Airflow Distribution

**Figure 7:**
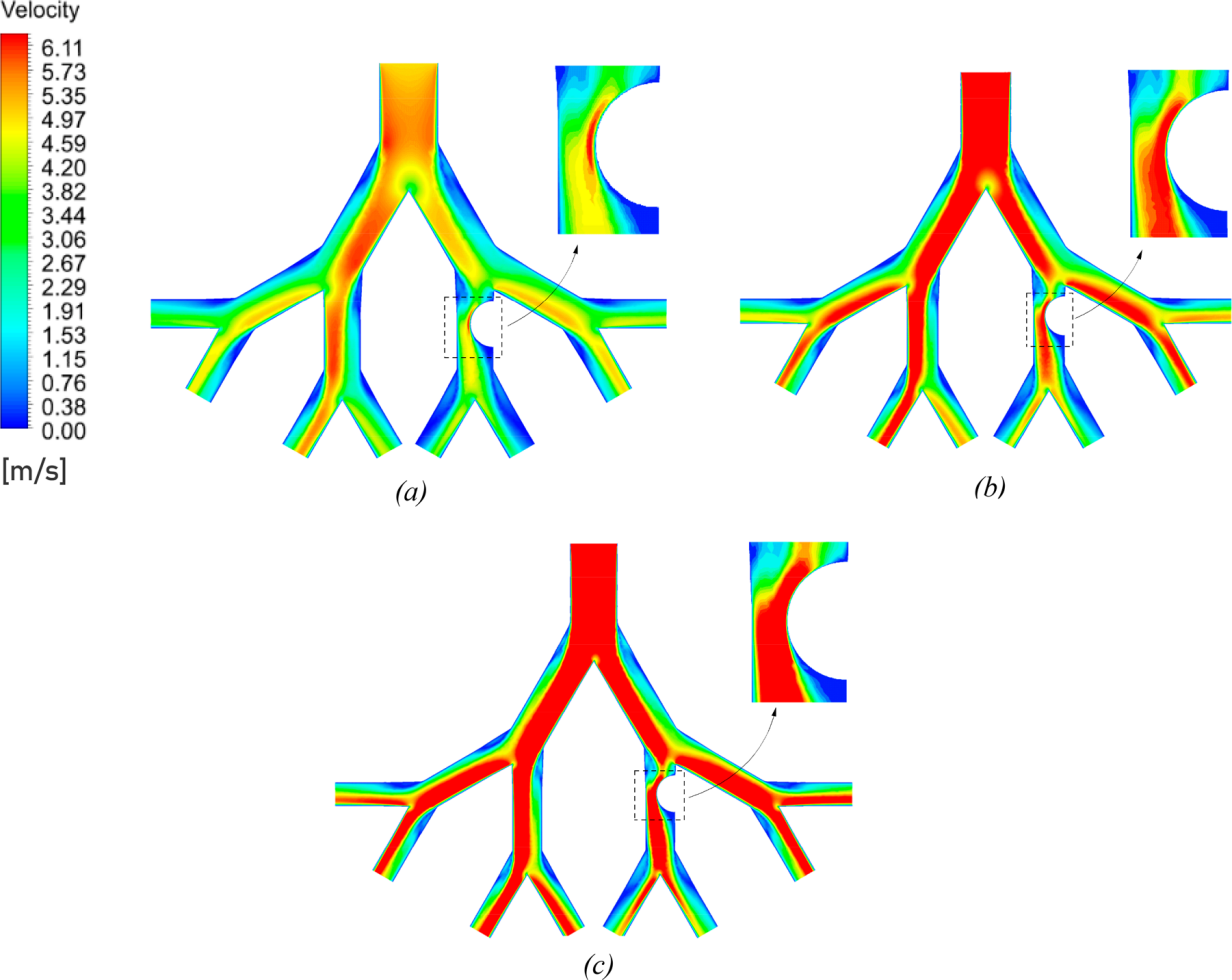
Airflow velocity contours on the symmetric plane for 50-year- (a), 60-year- (b), and 70-year-old (c) models at a flow rate of 60 L/min.

**Figure 8:**
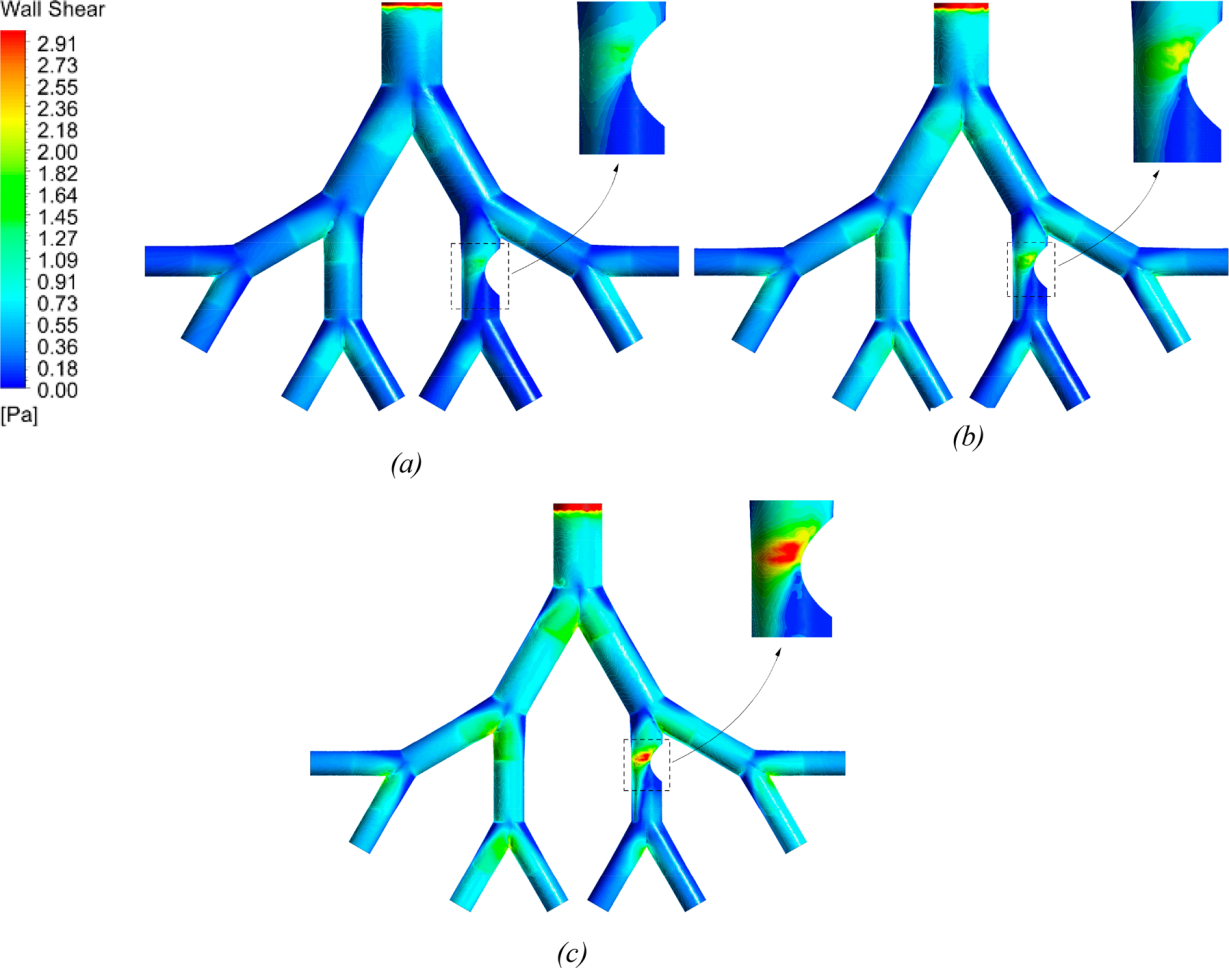
Wall shear contours for 50-year- (a), 60-year-, (b) and 70-year-old (c) models at a flow rate of 60 L/min.

**Figure 9:**
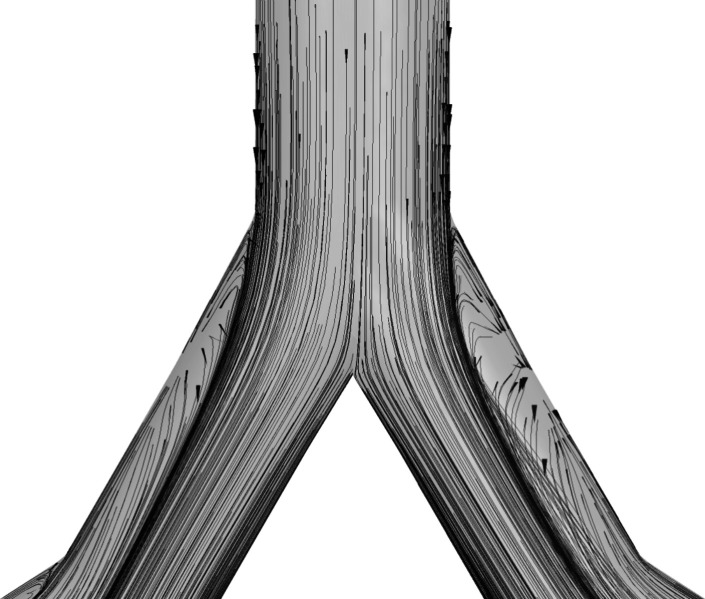
Separation of streamlines and generation of secondary vortices on the symmetric plane at the bifurcation region of G3.

The air velocity and wall shear stress contours for G3–G6 lung models of three ages are illustrated in Figures 7 and 8, respectively, for an inlet flow rate of 60 L/min. The contours show that velocity reduces as the flow moves into deeper generations because the total cross-sectional area of the airways increases. The velocity in the 70-year-old lung model is highest owing to its smallest diameter as compared to the other ages. The variation in velocity and wall shear at every bifurcation area is significant, and a trend of increased velocity and shear stress is found as the air passes through the bifurcation point. From the velocity contours in Figure 7, a nonuniform distribution of velocity as the flow passes the first and second bifurcation is found. As the air reaches the bifurcating point of the airways, its velocity skews toward the inner wall of the airway and reduces toward the outer wall. This is due to the separation of the streamlines owing to geometrical deflections at the bifurcation regions of the airways and the generation of secondary vortices as depicted in Figure 9. Moreover, the velocity and wall shear stress on the tumor increases with the increase of age. The impact mechanism indicates a higher particle deposition on the tumor wall and bifurcation point of the 70-year-old model and a low number of deposited particles for the 50-year-old model. The impact mechanism involves particles colliding with surfaces due to their inertia and is affected by both flow velocity and particle size.

**Figure 10:**
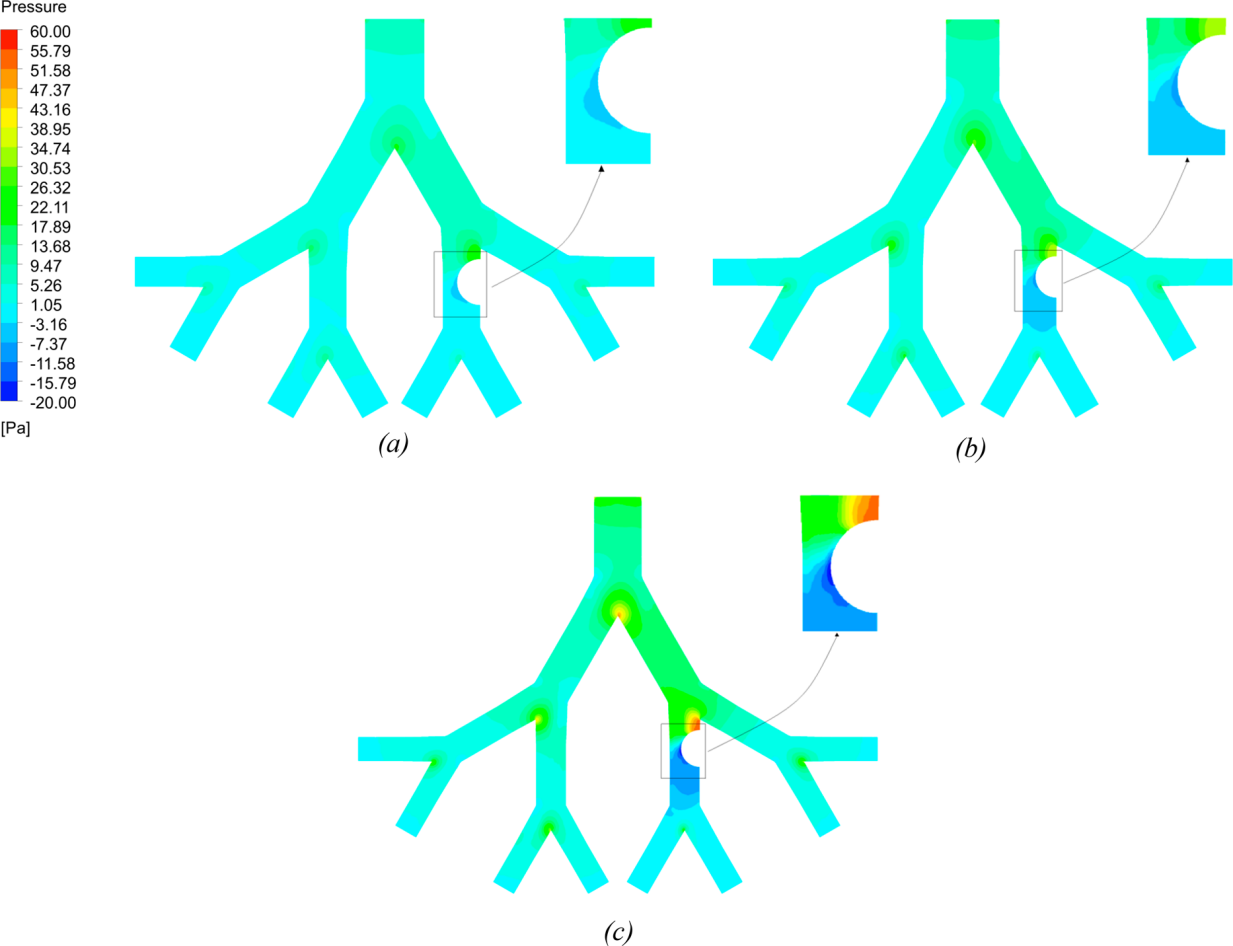
Pressure contours for 50-year- (a), 60-year- (b), and 70-year-old (c) models at a flow rate of 60 L/min.

The pressure contours for the three ages are shown in Figure 10 at an inlet flow rate of 60 L/min. The magnitude of pressure reduces as the flow moves into the deeper airways. As shown in Figure 7, a high velocity in the 70-year-old lung requires a high-pressure gradient to move the flow at the inlet. High pressure is found in the airway located before the unhealthy generation for all ages. This is because most of the flow gets skewed toward the left branch of the lung as it passes through the bifurcation point, leaving the right branch with low velocity and hence high pressure. Moreover, the magnitude of the pressure is found to be negative as the flow passes the constriction region. The narrow airway due to the tumor causes the air velocity to increase significantly, and as a result, the pressure in this region decreases.

**Figure 11:**
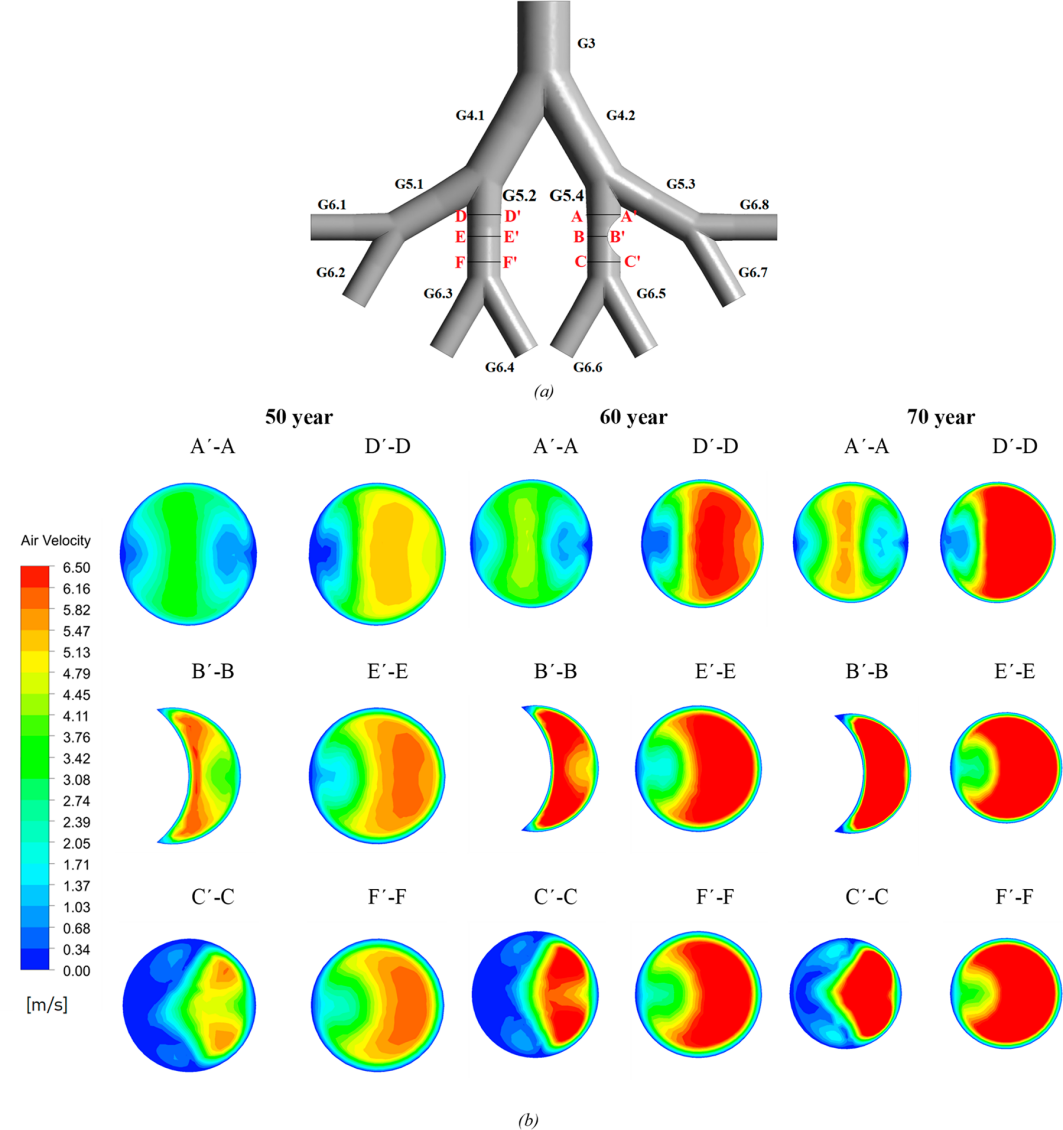
Location of cross-sectional planes in a lung model (a) and velocity contours at the different cross-sectional planes of 50-, 60-, and 70-year-old lung models for a flow rate of 60 L/min (b).

**Figure 12:**
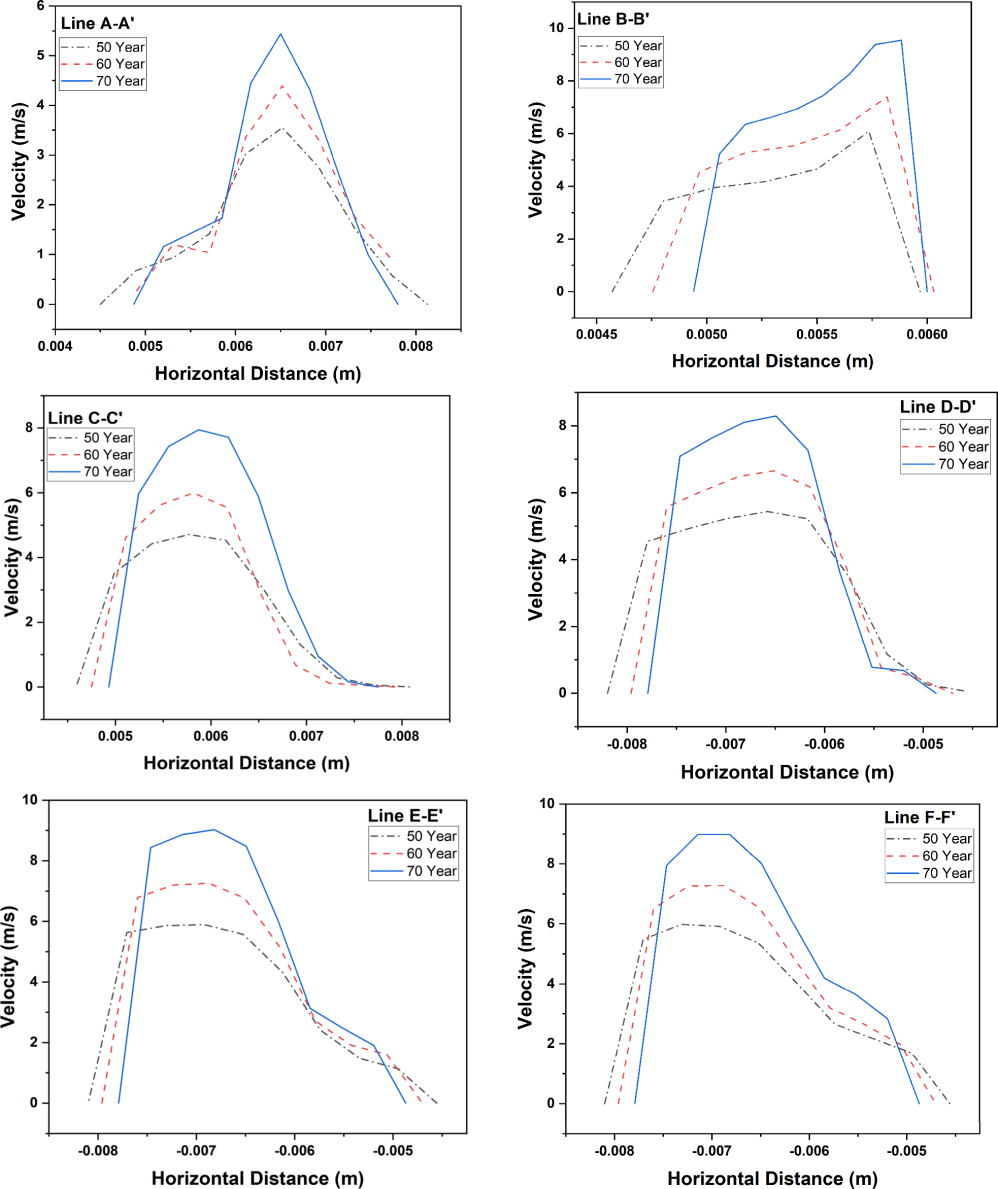
Comparison of the horizontal velocity of inhalation flows in the airways in relation to a developing bronchial tumor and healthy airways for each age lung model.

The velocity contours at the different cross-sectional planes defined in Figure 11a are shown in Figure 11b. These planes include those near the airway with a tumor and those near the airway at the same generation without a tumor. The flow moves from the inlet surface of G3 to the first bifurcation, where it divides into two main branches. However, the right branch of the lung model is partially blocked in G5.4 by the tumor, causing a constricted side in comparison to the left branch. This results in the flow being skewed more toward the left branch (G4.1) of the lung model, generating high-velocity contours in the healthy airway as compared to the unhealthy airway. From Figure 11b, the velocity distribution upstream of the tumor is almost symmetric as compared to the healthy lung without a tumor. The maximum velocity is found in the 70-year-old lung because the maximum shrinkage of the lung generations and the airflow patterns have considerable effects downstream from the tumor as compared to upstream. Also, the magnitudes of velocity in G5.2 with no tumor are higher, especially for the downstream plane when compared with the magnitudes of velocity in G5.4 with the presence of a tumor. Figure 12 illustrates the velocity distributions at horizontal lines located at the center of cross-sectional planes of each lung model at a flow rate of 60 L/min. The velocity gradient increases continuously as the age increases. It is interesting to observe that the velocity distribution remains almost symmetric along the line A–A′, whereas, for the line C–C′, the airflow velocity is skewed toward the inside of the airway, as is also found in Figure 11b. For the healthy generation, the velocity profile remains almost identical along the airway as indicated by the profiles of three lines, D–D′, E–E′, and F–F′.

**Figure 13:**
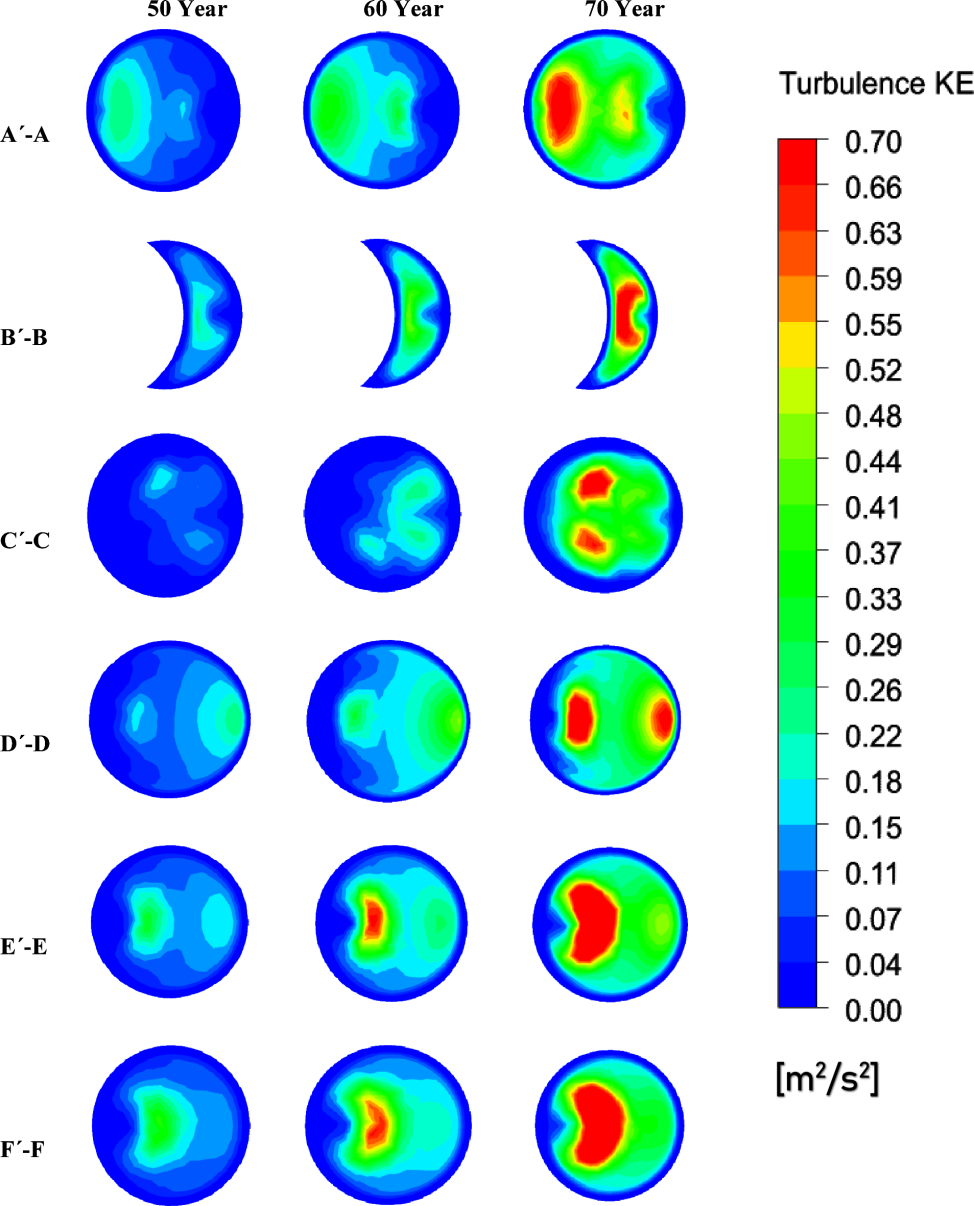
Turbulence kinetic energy at various sections of the tumor in each age model for a flow rate of 60 L/min.

Figure 13 displays the contours of turbulence energy across various cross-sectional planes located in the healthy and unhealthy airways at a flow rate of 60 L/min. The turbulence kinetic energy amplifies with age as the diameter of the airways narrows and flow velocity surges. Additionally, the highest level of turbulence kinetic energy occurs at the cross-sectional plane situated at the tumor’s center (B′–B) in all age models. The tumor causes disturbances in the airflow pattern within the airways, resulting in fluctuations during the inhalation process, ultimately leading to heightened turbulence energy at the central plane. When comparing the age models, maximum turbulence energy is found in the 70-year-old lung model because of a high velocity gradient and low airway diameters.

### 4.2 Deposition of Particles

**Figure 14:**
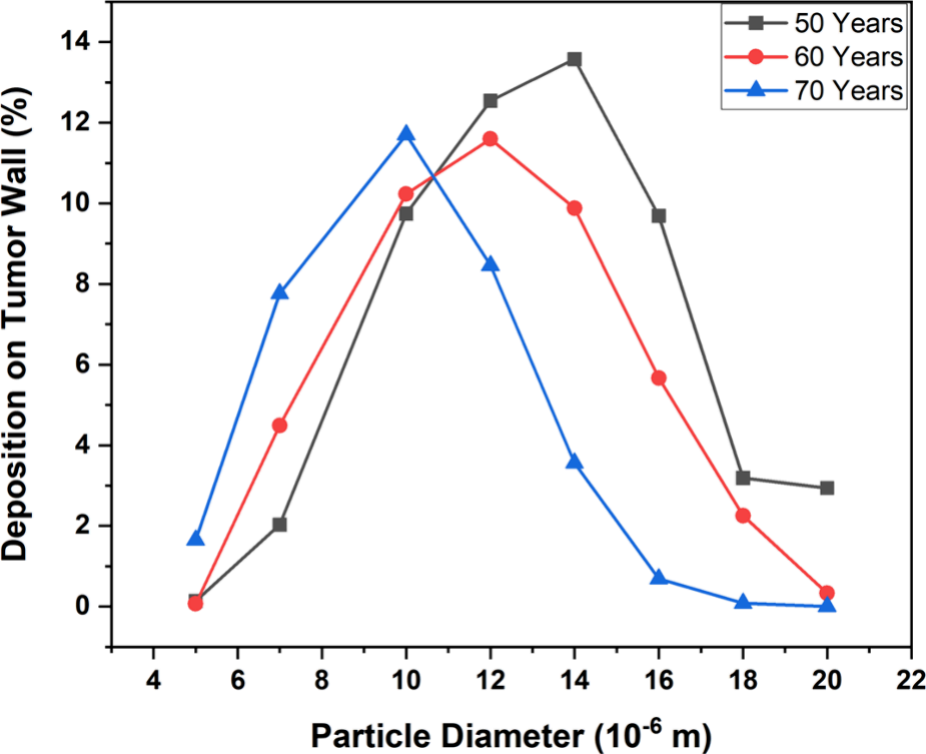
Deposition efficiency on the tumor wall for different ages and injected particle diameter at a flow rate of 60 L/min.

Figure 14 shows the variation of the deposition efficiency on the tumor wall for three ages. The deposition efficiency reaches its maximum when the particle diameter is 10, 12, and 14 μm for 70-, 60-, and 50-year-old models, respectively. For very small particles, the deposition efficiency is the lowest because the impact mechanism for small-sized particles becomes weak. As the flow splits at the bifurcation point, the small particles can alter their direction and can conveniently follow the flow movement, which makes it easy for them to escape the upper generations and deposit in the lower bronchioles of the lung. For the large particle size, the impact mechanism is strong, which makes the inhaled aerosols hit the boundary wall and deposit at the upper bifurcation point. These particles do not immediately follow the changes in the flow directions, and this makes it difficult for the large particles to deposit in the lower generations. For the present case, the small particles (<10 μm) mostly escaped from generations G3–G6 to deposit in the lower generations, whereas the large particles (>14 μm) got deposited mostly in G3 and G4 before reaching the tumor wall in G5.

For particle sizes ranging from 5 to 10 μm, the deposition efficiency in the 50-year-old lung model was comparatively less due to lower inlet velocity and large surface area. The majority of these particles escaped the lung model to deposit in the deeper generations. But, for large-size particles, the maximum deposition occurred in generations G3–G6, and the maximum deposition on the tumor wall is found for a particle size of 14 μm in the 50-year-old lung model. Particles greater than 14 μm mostly got trapped in the regions above the tumor wall, and hence, the deposition efficiency decreased for particles with a size greater than 14 μm. A similar trend was found for the 60-year-old lung model, which has a maximum deposition on the tumor wall for a particle size of 12 μm. The airways in the 60-year-old lung model were narrower than in the 50-year-old lung model, and the large particles with a size greater than 12 μm were more vulnerable to getting trapped in the upper generations of the lung before reaching the tumor region. Hence, the maximum deposition efficiency for the 60-year-old lung model was less and occurred for a lower particle size than the 50-year-old lung model. Compared to the 50- and 60-year-old models, the deposition on the tumor wall of the 70-year-old lung model was highest for a particle size range of 5–10 μm. The maximum deposition efficiency for the 70-year-old lung model was found for a particle size of 10 μm. For particle sizes larger than 10 μm, the deposition efficiency started to decrease as the majority of the large-size particles got deposited in the narrow airways of upper generations before reaching the tumor wall.

**Figure 15:**
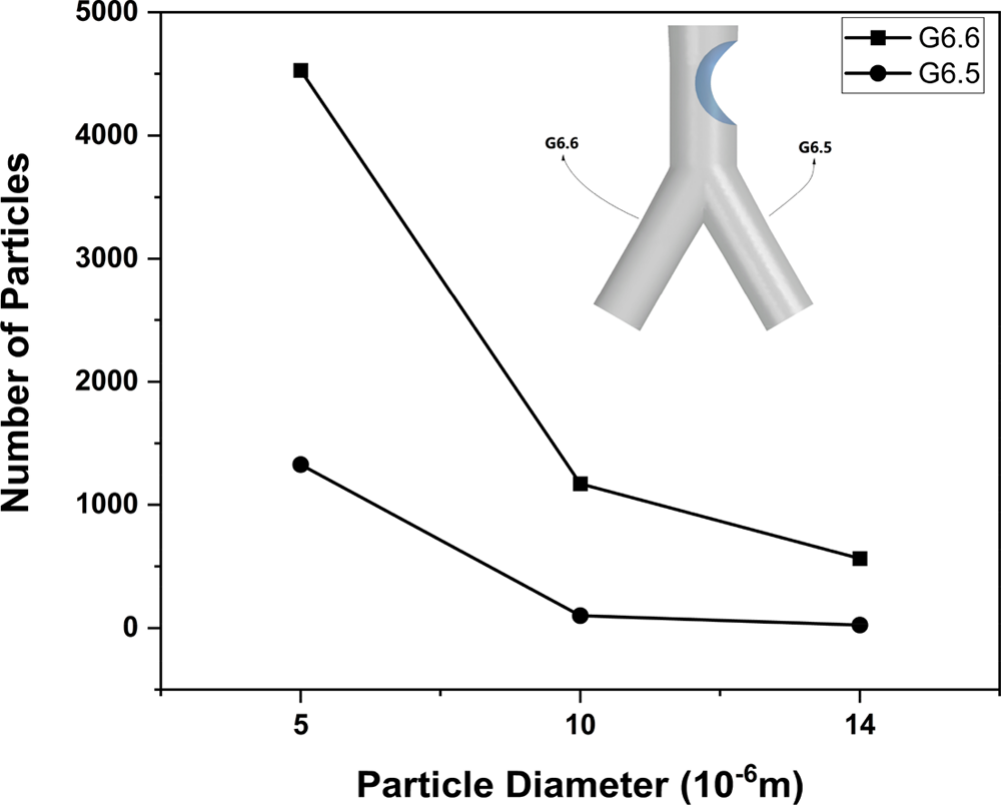
Comparison of number of injected particles traveling in the branches following the tumor.

The presence of a tumor affects the transport of the injected particles. The tumor present in G5 on one side of the wall influences the flow of the traveling particles and diverts them toward the opposite side of the airway. Figure 15 demonstrates the comparison between the percentage of injected particles for three different particle sizes moving into both branches following the airway with the tumor. The number of particles traveling toward G6.6 is higher than that toward G6.5 for each particle size due to the presence of the tumor on the same side as in G6.5 in G5. This causes uneven particle flow distribution and deposition efficiency in the branches following the airway with a tumor.

**Figure 16:**
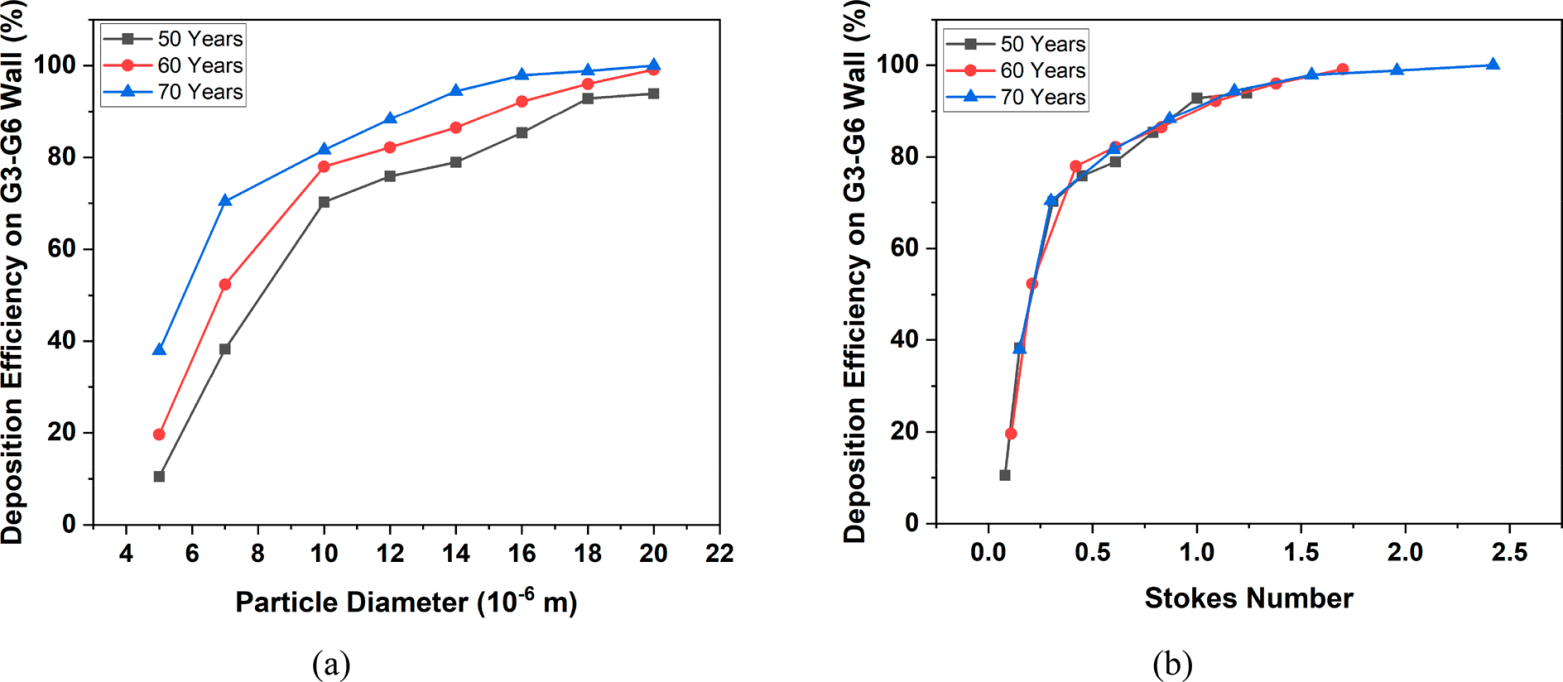
Deposition of particles on the G3–G6 wall against particle diameter (a) and Stokes number (b) at a flow rate of 60 L/min.

Figure 16a and b show the deposition efficiency of particles in the lung wall other than the tumor against particle diameter and Stokes number, respectively. For small particles (5 μm), the deposition efficiency on the G3–G6 wall is 10.52%, 19.63%, and 37.95% for 50-, 60-, and 70-year-old lung models, respectively. The majority of the small particles escape the G3–G6 section of the lung. As the particle size increases, the deposition rate increases, whereas the escape rate decreases. In the case of large-sized particles (20 μm), the escape rate is almost negligible, as all the particles are deposited on the upper generations of the lung model. Only for the particles belonging to a 10–14 μm range, is the deposition on the tumor wall considerable for each age. A similar trend of variation of deposition efficiency for small and large particle sizes is found against the Stokes number in Figure 16b. For each age, the Stokes number varies as it depends on both the inlet velocity at the G3 and the airway diameter. As age progresses, the diameter of the conducting airways decreases which leads to a higher velocity and turbulence. Consequently, a higher Stokes number is found at which the deposition of inhaled particles on the lung wall increases significantly in the upper generations.

**Figure 17:**
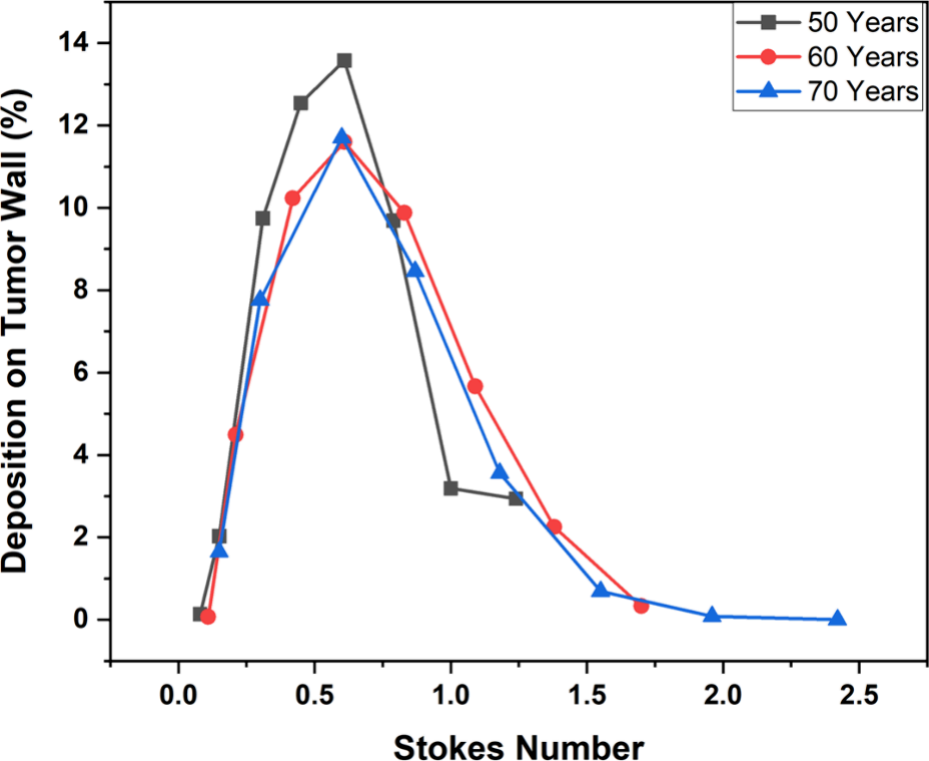
Variation of deposition efficiency on the tumor wall with age and Stokes number for the 60 L/min flow rate.

Figure 17 illustrates the deposition on the tumor wall against different stokes numbers and ages. It shows that due to a higher Stokes number, the majority of the injected particles get trapped in the walls of the upper generation and a small number of particles were only deposited on the tumor wall in G5. A higher Stokes number in older age also ensures that the deposition of particles on the G3–G6 lung wall of the 70-year-old is higher than its younger counterparts (60 and 50 years). Whereas, for the tumor wall, the maximum deposition efficiency for age 50-, 60- and 70-year-old lung models is 13.57%, 11.59%, and 11.70% respectively in the diameter range of 10 to 14 μm for the injected particles.

**Figure 18:**
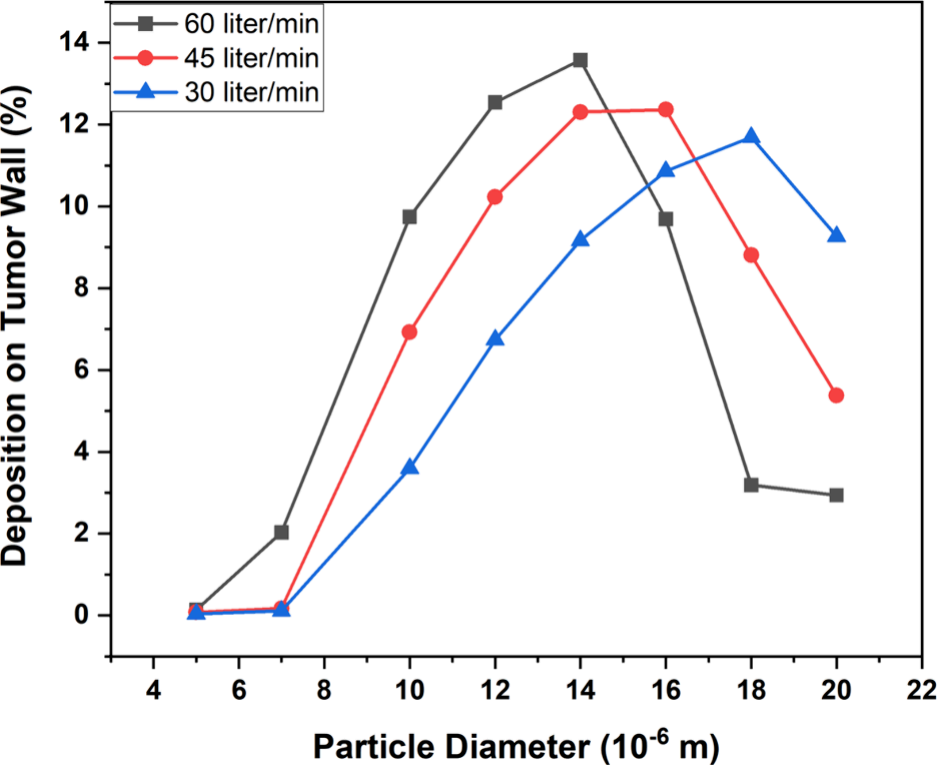
Deposition efficiency on the tumor wall for different inlet flow rates and injected particle diameter for the 50-year-old lung model.

**Figure 19:**
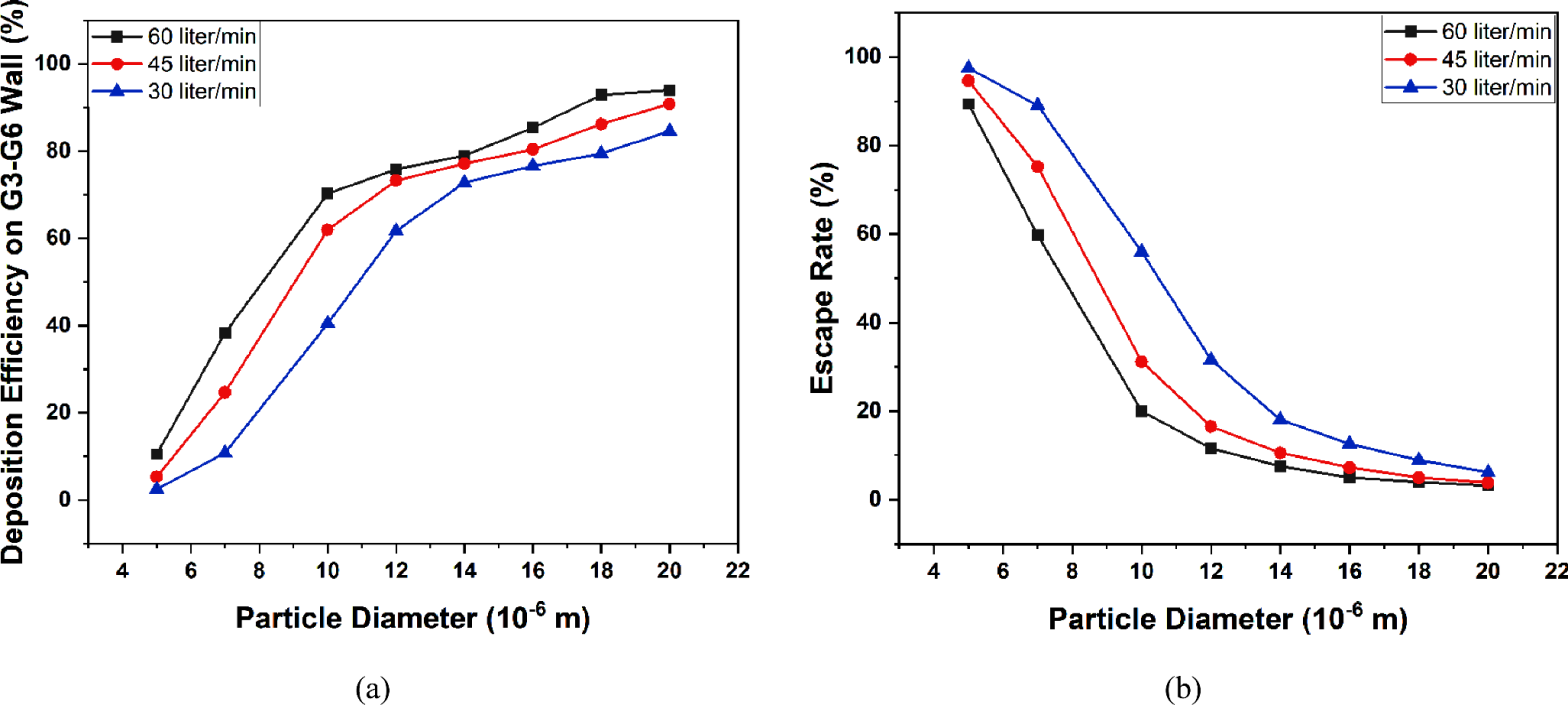
Deposition of particles on the G3–G6 wall (a) and escape rate (b) for different flow rates and diameter sizes for the 50-year-old lung model.

The effect of various inhalation conditions is also investigated for a 50-year-old lung model by using three different inlet air flow rates, specifically 60 L/min, 45 L/min, and 30 L/min, which corresponds to heavy, moderate, and light breathing, respectively. The deposition efficiency on the tumor wall is at a maximum for a 60 L/min flow rate followed by 45 L/min and 30 L/min, as shown in Figure 18. The deposition efficiency on the tumor wall is less for the small inlet flow rate because of low inertial forces and a weak impact mechanism. The deposition rate increases with the high inlet flow rate. For each flow rate, the drug deposition efficiency on the tumor wall can be improved by increasing the size of the injected particle, but there is a limit to this condition as well. For large and moderate inlet flow rates, a particle size greater than 14 and 16 μm, respectively, causes the trapping of particles on the tumor wall to reduce significantly as most of the particles are already deposited in the upper generations before reaching the tumor wall. For a small inlet flow rate, the particle deposition for a large particle size (18 μm) is much higher than the small-sized particles but still lower than the other inlet flow rates. This is because, due to the small velocity and approximately streamlined flow for a small inlet flow rate, particles tend to move along straight path lines and travel through the upper generations without depositing and trapping deeper into the lungs such as on the tumor wall present in G5. The maximum deposition efficiency on the tumor wall was 13.57%, 12.36%, and 11.69% for large, moderate, and small inlet flow rates, respectively. Also, the particle deposition on the lung walls is higher for a large inlet flow rate as depicted in Figure 19a. The escape rate for small inlet flow rate is higher compared to those for moderate and large flow rates (see Figure 19b).

**Figure 20:**
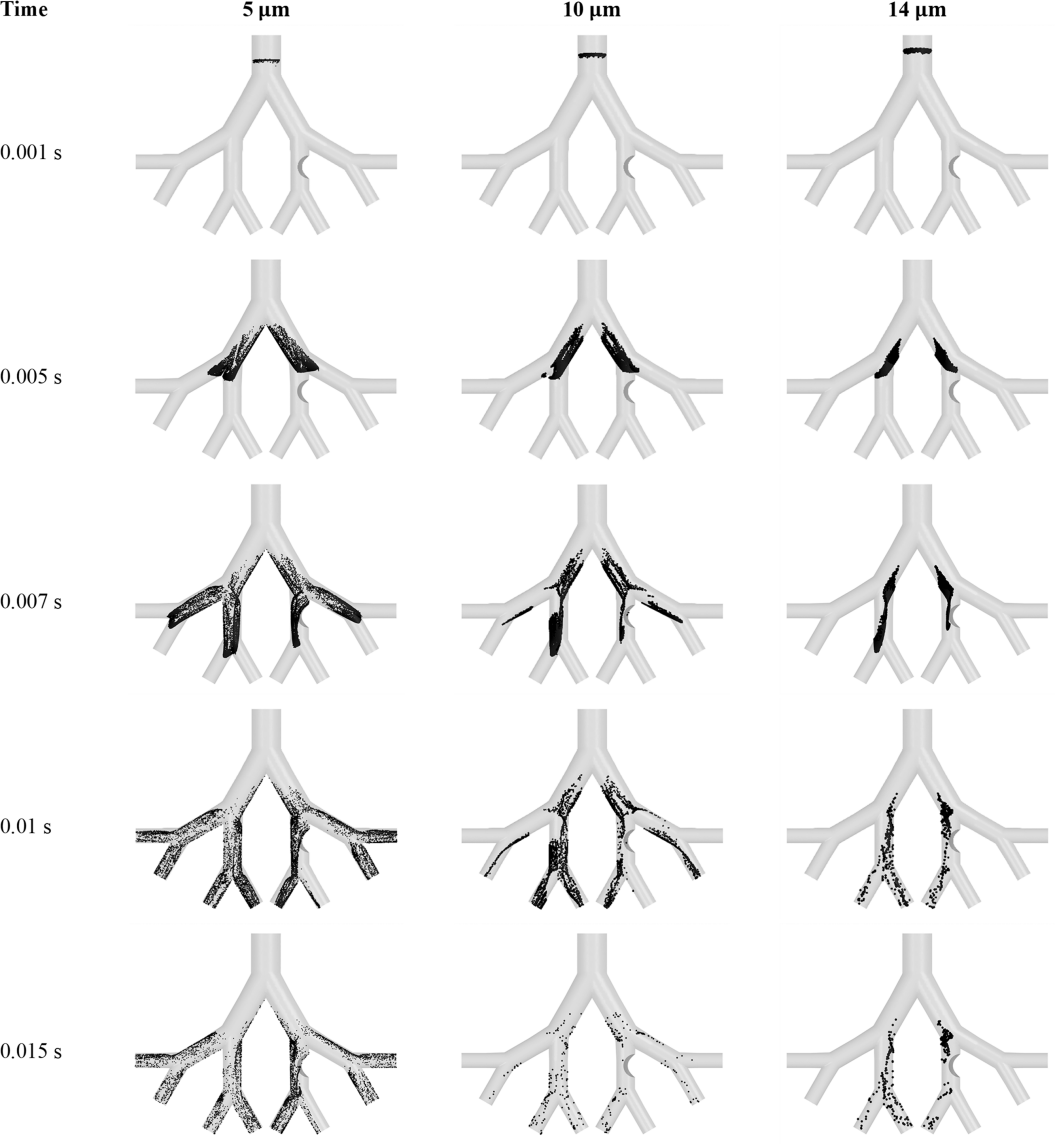
Particle transport in the 50-year-old lung model for particle sizes of 5, 10, and 14 μm at a flow rate of 60 L/min.

Figure 20 illustrates the transport of particles with sizes 5 μm, 10 μm, and 14 μm in the 50-year-old lung model for an inlet flow rate of 60 L/min. The particle transport is demonstrated at different timesteps to visualize the location and flow behavior of particles through the G3–G6 lung model. As demonstrated in Figure 20, a higher concentration of particles is obtained in the domain of the lung model for a particle size of 5 μm, indicating a lower deposition efficiency on the G3–G6 and tumor wall. However, the deposition of particles with a size of 14 μm is the maximum for the 50-year-old lung model, which can be indicated by the low concentration of particles in the domain of the lung model for a particle size of 14 μm. For the present study, the optimal size is found to be in the range of 10–14 μm for maximum effectiveness of the inhaled drug. Also, small-sized particles move into the deeper airways, whereas the large-sized particles just deposit even before reaching the targeted region for each inlet flow rate.

**Figure 21:**
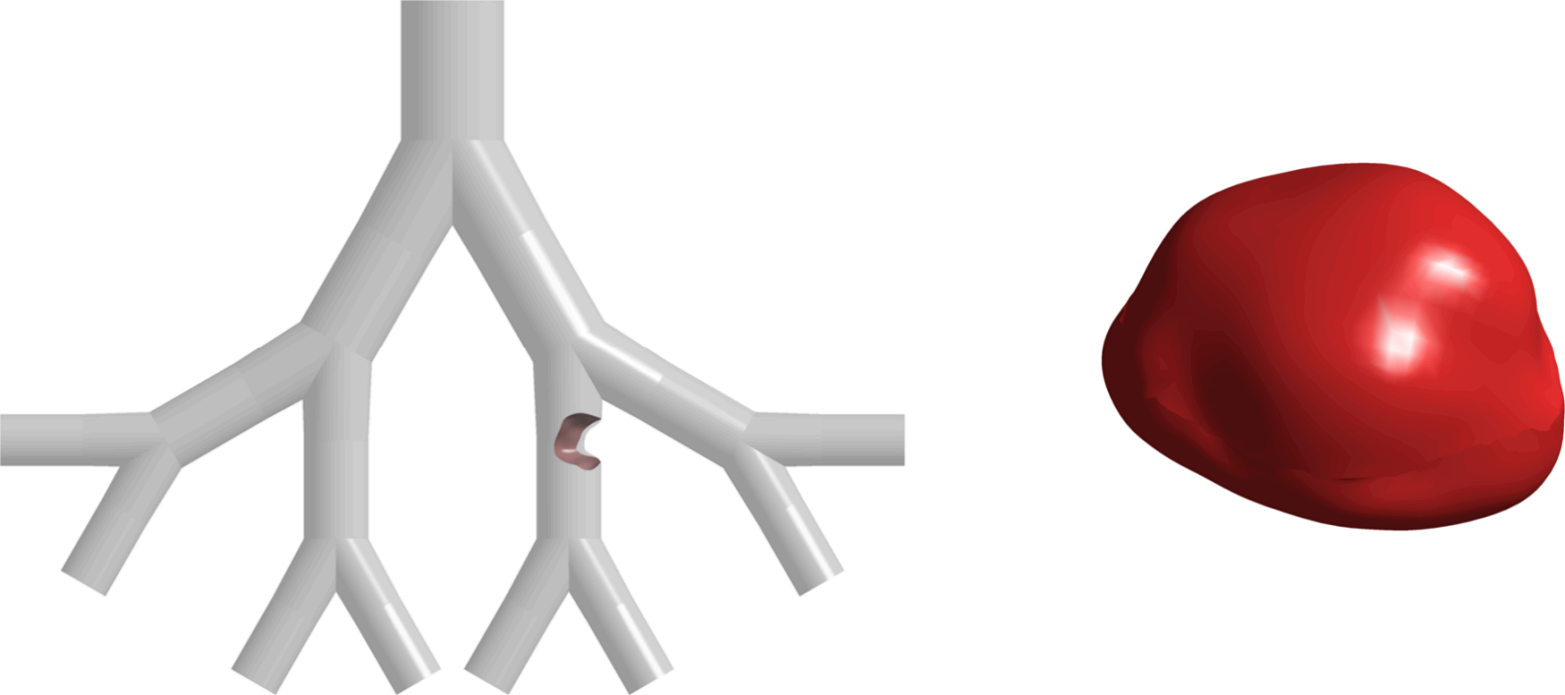
Real lung tumor placed in a G3–G6 lung model for a 50-year-old example.

**Figure 22:**
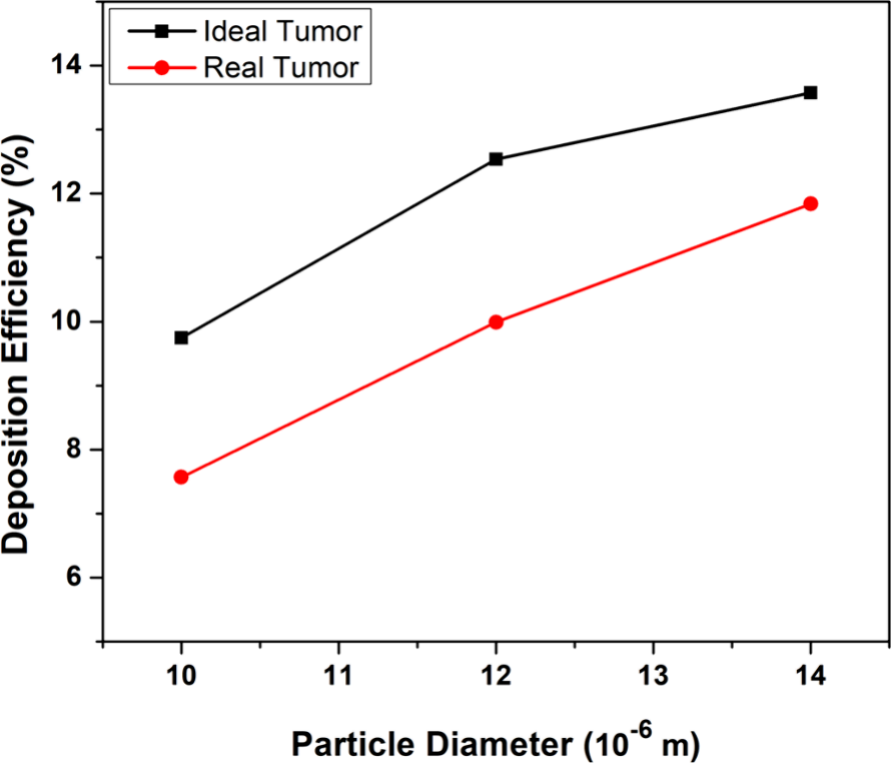
Deposition efficiency for ideal and real tumor shape at an inlet flow rate of 60 L/min.

So far, the analysis has been conducted by inserting an ideal tumor shape at a single location in the airways. However, studying the effects of a real tumor shape and locating the tumor at different positions within the airways is also important to understanding the variations in airflow patterns and deposition rates. Figure 21 illustrates a tumor that mimics the real shape of a lung tumor,^33,60^ is placed in a similar location, and blocks a similar percentage of airway as the ideal tumor. When the particle sizes with the highest deposition efficiencies in the case of the ideal tumor model were injected into the model with the real tumor, a slight decrease was found. This can be because the real tumor, even though blocking a similar percentage of the airway as the ideal tumor, experienced fewer particle collisions due to an uneven surface profile. The comparison of deposition efficiencies for both ideal and real tumor shapes is shown in Figure 22.

**Figure 23:**
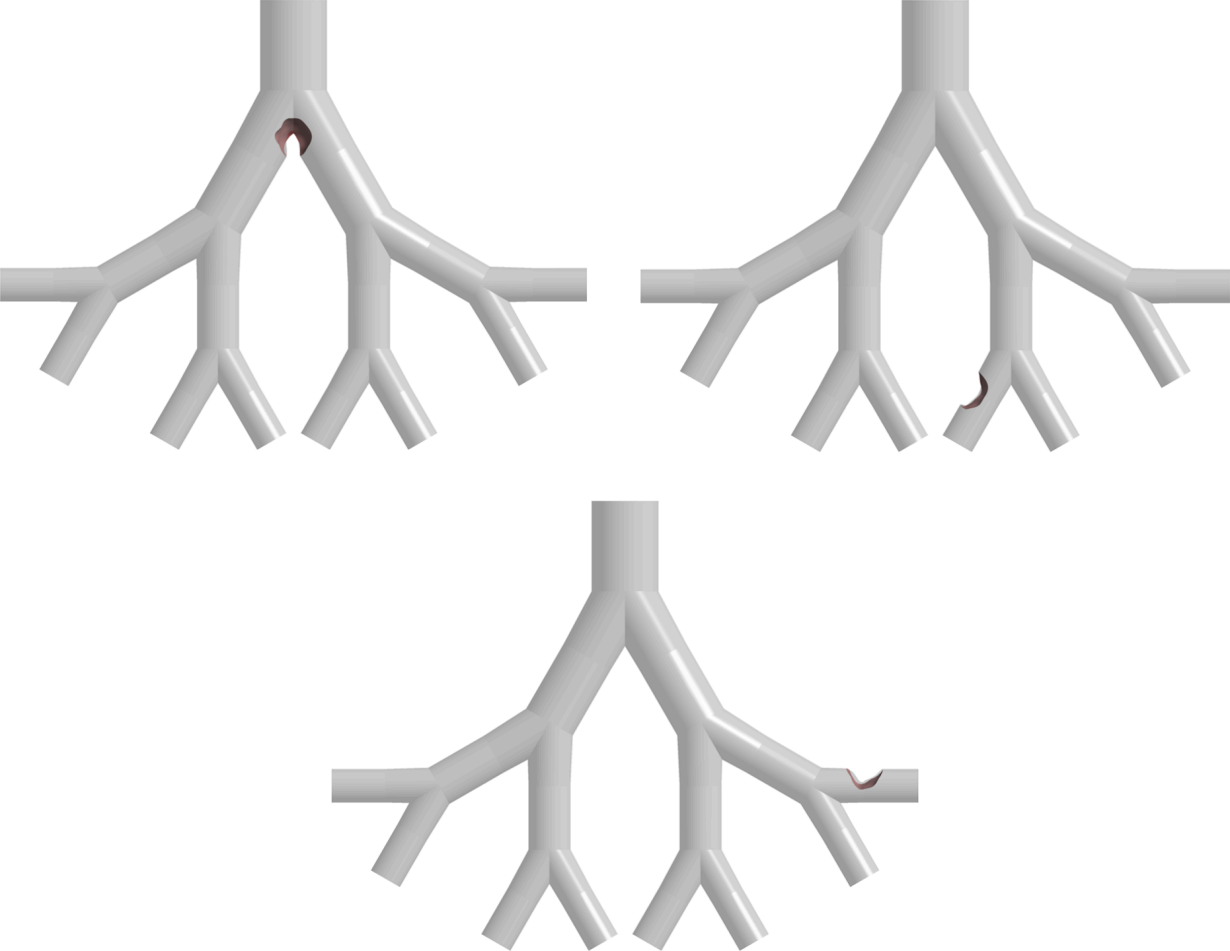
Models with different tumor locations.

**Figure 24:**
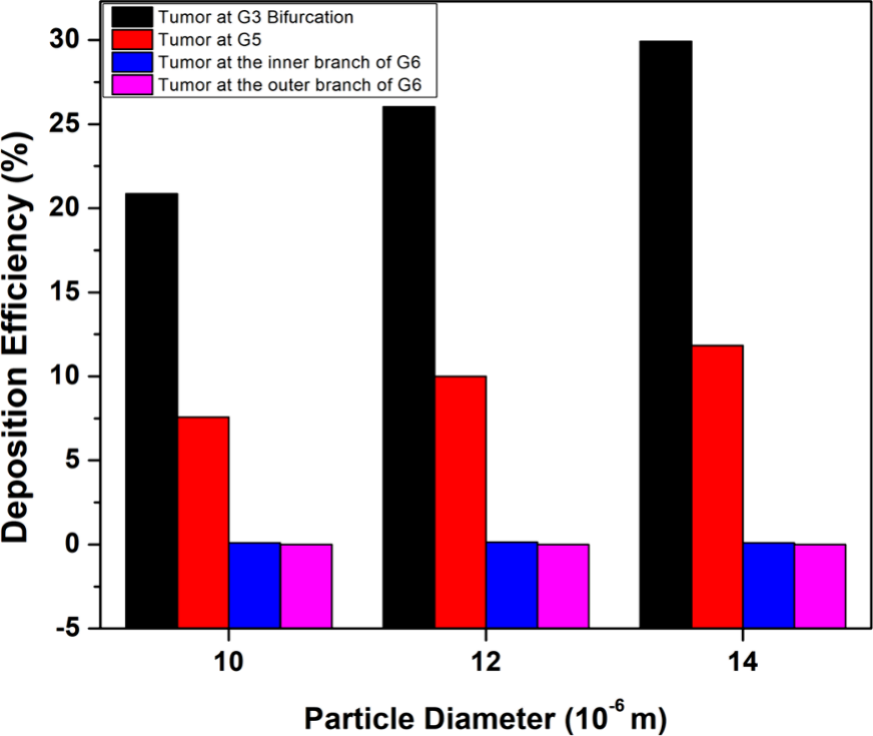
Deposition efficiencies for different lung models at 10, 12, and 14 μm particle sizes.

**Figure 25:**
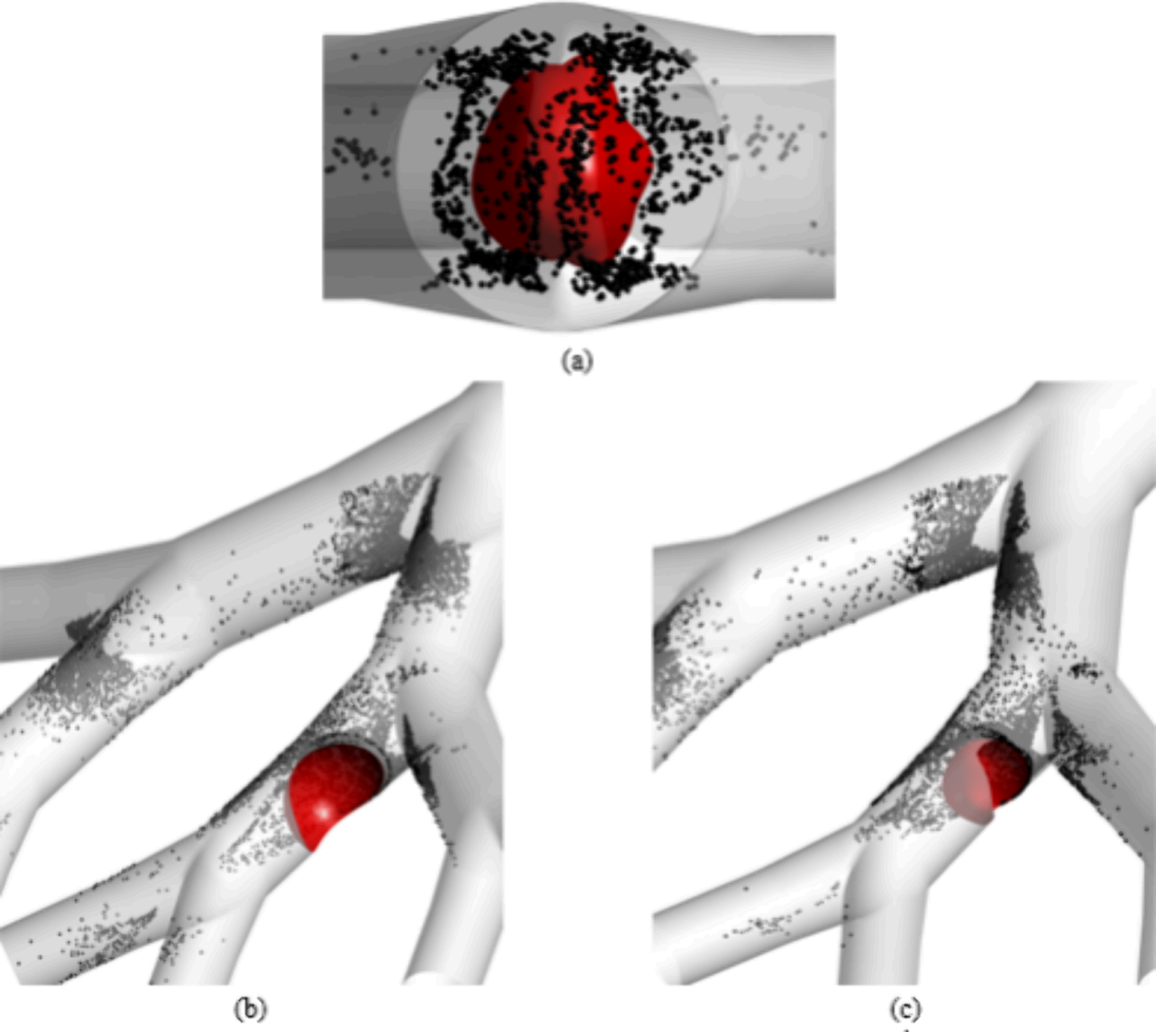
Local particle deposition on (a) a real tumor at G3 bifurcation, (b) an ideal tumor at G5, and (c) a real tumor at G5.

The location of the tumor is also varied to understand its influence on particle deposition. Figure 23 illustrates three models, each with different tumor locations: a tumor at the G3 bifurcation, a tumor at the inner branch of G6, and a tumor at the outer branch of G6. The location of the tumor in the airways significantly impacts its treatment procedures and can facilitate healthcare experts in developing a personalized treatment plan for the patient. Tumors at different locations experience varying particle collisions, and identifying the optimal particle size and inhalation velocity, depending on tumor locations, for maximum drug effectiveness is crucial for optimized treatment. Figure 24 shows the difference in deposition rates for each of the tumor locations. The deposition efficiency at the G3 bifurcation tumor is at a maximum since this region experiences direct particle collisions. On the other hand, for tumors present in the G6 generation, the deposition efficiency is negligible for the given particle sizes. This is because the majority of these particle sizes are optimal for depositing in the upper generation and do not go deeper into the G6. To deposit more particles at the surface of such tumors present in G6, a suitable range of particle sizes, along with advanced deposition techniques such as magnetic drug targeting (MDT),^61^ is preferable. The local particle deposition at a flow rate of 60 L/min and for a particle size of 10 μm is demonstrated for different tumor shapes and locations in Figure 25.

## 5 Conclusions

The TD of microscale particles in the tracheobronchial airway generations G3–G6 of an unhealthy lung with a presence of a sidewall tumor for three sets of ages and three inlet air flow rates is investigated. Wall shear stress, velocity contours at different cross-sectional planes, and pressure contours are compared for each lung model and the key findings from the study are summarized below:

- As the age increased, the airflow velocity on the tumor wall also increased due to changes in the geometrical parameters of the lung. The velocity, wall shear, and pressure drop increased with the inlet flow rate for each age.
- Due to increased velocity magnitudes and the impact mechanism for older age, the particle deposition on the G3–G6 lung wall for the 70-year-old lung is the maximum when compared with the 60- and 50-year-old lung models. Moreover, for the tumor wall, a 70-year-old lung has the highest deposition efficiency for a particle size up to 10 μm, and for a particle size ranging between 10 and 20 μm, the maximum deposition efficiency is found in the 50-year-old lung.
- The presence of a tumor can significantly alter the trajectory of incoming particles, resulting in a diversion toward the opposite side of the airway. Consequently, a nonuniform distribution of particles occurs in the generation following the obstructed airway, leading to one branch receiving a lower particle count than the other.
- The deposition of particles on the tumor wall depends greatly on the size of the injected particles. Particles with a 10–14 μm diameter have maximum deposition on the tumor wall located in the fifth generation for each age, whereas particles with a size less than 10 μm mostly escape the G3–G6 lung region and are deposited into the deeper airways. For particles with a size greater than 14 μm, the deposition efficiency is higher in the upper generations, and only a few injected particles reach the tumor wall. Also, the variation trends of deposition efficiency with respect to particle size for the tumor wall and G3–G6 lung wall are different. Hence, depending on the location of the targeted region inside an age-specific lung, the size of the injected particle can be adjusted for optimal delivery and treatment.
- The inlet flow rate also has a significant effect on the deposition efficiency. More particles are deposited on the tumor wall for a large inlet flow rate as compared to particles of the same size for medium and small inlet flow rates for the same age.
- Moreover, for a small inlet flow rate, particles with large diameters tend to move deeper into the airways as compared to the large inlet flow rate. Hence for a large flow rate, particle sizes ranging between 10 and 14 μm, and for a small inlet flow rate, particle size ranging between 12 and 18 μm, are optimal for treating the growth of a glomus tumor in the upper tracheobronchial airways.
- The comparison between ideal and real tumor shapes showed a slight difference between particle deposition efficiencies. Additionally, it is important to note that the location of the tumor plays a critical role in terms of particle deposition under the same inhalation conditions.

The present study critically analyzed the flow behavior in a tumorous lung, aiming to understand its implications for human health and well-being. Comprehensive qualitative and quantitative analysis of the velocity magnitude, pressure drop, and wall shear would help to understand the breathing dynamics of cancer patients. The detailed analysis of aerosol transport dynamics through the tumorous section of the airways improves the knowledge of the field and helps the development of future therapeutics. The findings of the present study would be beneficial for enhancing the quality of drug delivery through inhaling equipment and for better treatment of pulmonary diseases. For future studies, the employment of realistic respiratory models based on the computed tomography scans of real patients is recommended for more comprehensive analysis.

### 5.1 Limitations of the Study

Limitations of the study are as follows:

- An idealized airway model used as patient-specific model is not available for validation.
- Open outlet and uniform pressure are used at the outlet.
- The study only considered inhalation.

## Data Availability

Data will be available upon reasonable request.

## References

[ref1] Cohen A J Outdoor air pollution and lung cancer. Environ. Health Perspect. 2000, 108 (suppl 4), 743–750. 10.1289/ehp.00108s4743. PMC1637685 10931793

[ref2] Akhtar N.; Bansal J. G. Risk factors of Lung Cancer in nonsmoker. Current Problems in Cancer 2017, 41 (5), 328–339. 10.1016/j.currproblcancer.2017.07.002. 28823540

[ref3] Kleinstreuer C.; Zhang Z. Targeted drug aerosol deposition analysis for a four-generation lung airway model with hemispherical tumors. Journal of Biomechanical Engineering 2003, 125 (2), 197–206. 10.1115/1.1543548. 12751281

[ref4] Alberg A. J.; Samet J. M. Epidemiology of lung cancer. Chest 2003, 123 (1), 21S–49S. 10.1378/chest.123.1_suppl.21S. 12527563

[ref5] Hohenforst-Schmidt W.; et al. Glomus tumor in the lung parenchyma. Journal of Thoracic Disease 2012, 4 (6), 663 10.3978/j.issn.2072-1439.2012.10.01. 23205298 PMC3506793

[ref6] Sung H.; Ferlay J.; Siegel R. L.; Laversanne M.; Soerjomataram I.; Jemal A.; Bray F. Global cancer statistics 2020: GLOBOCAN estimates of incidence and mortality worldwide for 36 cancers in 185 countries. CA: Cancer J. Clinicians 2021, 71 (3), 209–249. 10.3322/caac.21660. 33538338

[ref7] Oliveira R. F.; Teixeira S. F.C.F.; Silva L. F.; Teixeira J. C.F.; Antunes H. Development of new spacer device geometry: a CFD study (part I). Computer Methods in Biomechanics and Biomedical Engineering 2012, 15 (8), 825–833. 10.1080/10255842.2011.563359. 21491261

[ref8] Borghardt J. M.; Kloft C.; Sharma A. Inhaled therapy in respiratory disease: the complex interplay of pulmonary kinetic processes. Canadian Respiratory Journal 2018, 2018, 1 10.1155/2018/2732017. PMC6029458 30018677

[ref9] Valerian Corda J.; Emmanuel J.; Nambiar S.; K P.; Zuber M. Airflow patterns and particle deposition in a pediatric nasal upper airway following a rapid maxillary expansion: Computational fluid dynamics study. Cogent Engineering 2023, 10 (1), 2158614 10.1080/23311916.2022.2158614.

[ref10] Inthavong K.; Ye Y.; Ding S.; Tu J. Y. Comparative study of the effects of acute asthma in relation to a recovered airway tree on airflow patterns. In 13th International Conference on Biomedical Engineering: ICBME 2008 3–6 December 2008 Singapore; Springer, 2009; pp 1555–1558.

[ref11] Huang F.; Zhu Q.; Zhou X.; Gou D.; Yu J.; Li R.; Tong Z.; Yang R. Role of CFD based in silico modelling in establishing an in vitro-in vivo correlation of aerosol deposition in the respiratory tract. Adv. Drug Delivery Rev. 2021, 170, 369–385. 10.1016/j.addr.2020.09.007. 32971228

[ref12] Gemci T.; Ponyavin V.; Collins R.; Corcoran T. E.; Saha S. C.; Islam M. S. CFD study of dry pulmonary surfactant aerosols deposition in upper 17 generations of human respiratory tract. Atmosphere 2022, 13 (5), 726 10.3390/atmos13050726.

[ref13] Islam M. S.; Larpruenrudee P.; Saha S. C.; Pourmehran O.; Paul A. R.; Gemci T.; Collins R.; Paul G.; Gu Y.; et al. How severe acute respiratory syndrome coronavirus-2 aerosol propagates through the age-specific upper airways. Phys. Fluids 2021, 33 (8), 081911 10.1063/5.0061627. PMC8450910 34552312

[ref14] April Si X.; Talaat M.; Xi J. SARS COV-2 virus-laden droplets coughed from deep lungs: Numerical quantification in a single-path whole respiratory tract geometry. Phys. Fluids 2021, 33 (2), 023306 10.1063/5.0040914. PMC7976054 33746489

[ref15] Rahman M.; Zhao M.; Islam M. S.; Dong K.; Saha S. C. Numerical study of nano and micro pollutant particle transport and deposition in realistic human lung airways. Powder Technol. 2022, 402, 117364 10.1016/j.powtec.2022.117364.

[ref16] Farkas A.; Furi P.; Then W.; Salma I. Effects of hygroscopic growth of ambient urban aerosol particles on their modelled regional and local deposition in healthy and COPD-compromised human respiratory system. Sci. Total Environ. 2022, 806, 151202 10.1016/j.scitotenv.2021.151202. 34736753

[ref17] Taheri M. H.; Pourmehran O.; Sarafraz M. M.; Ahookhosh K.; Farnoud A.; Cui X. Effect of swirling flow and particle-release pattern on drug delivery to human tracheobronchial airways. Biomechanics and Modeling in Mechanobiology 2021, 20, 2451–2469. 10.1007/s10237-021-01518-5. 34515918

[ref18] Jin Y.; Cui H.; Chen L.; Sun K.; Liu Z. Effects of airway deformation and alveolar pores on particle deposition in the lungs. Sci. Total Environ. 2022, 831, 154931 10.1016/j.scitotenv.2022.154931. 35364181

[ref19] Ou C.; Hang J.; Deng Q. Particle deposition in human lung airways: effects of airflow, particle size, and mechanisms. Aerosol and Air Quality Research 2020, 20 (12), 2846–2858. 10.4209/aaqr.2020.02.0067.

[ref20] Bhardwaj S.; Koullapis P.; Kassinos S. C.; Sznitman J. Fate of inhaled aerosols under the influence of glottal motion in a realistic in silico human tracheobronchial tree model. European Journal of Pharmaceutical Sciences 2022, 173, 106172 10.1016/j.ejps.2022.106172. 35351584

[ref21] Allon R.; Bhardwaj S.; Sznitman J.; Shoffel-Havakuk H.; Pinhas S.; Zloczower E.; Shapira-Galitz Y.; Lahav Y. A Novel Trans-Tracheostomal Retrograde Inhalation Technique Increases Subglottic Drug Deposition Compared to Traditional Trans-Oral Inhalation. Pharmaceutics 2023, 15 (3), 903 10.3390/pharmaceutics15030903. 36986764 PMC10056688

[ref22] Riaz H. H.; Lodhi A. H.; Munir A.; Zhao M.; Farooq U.; Qadri M. N. M.; Islam M. S. Breathing in danger: Mapping microplastic migration in the human respiratory system. Phys. Fluids 2024, 36 (4), 043338 10.1063/5.0205303.

[ref23] Segal R. A.; Guan X.; Shearer M.; Martonen T. B. Mathematical model of airflow in the lungs of children I; effects of tumor sizes and locations. Computational Mathematical Methods in Medicine 2000, 2 (3), 199–213. 10.1080/10273660008833046.

[ref24] Yang X.L.; Liu Y.; Luo H.Y. Respiratory flow in obstructed airways. J. Biomech. 2006, 39 (15), 2743–2751. 10.1016/j.jbiomech.2005.10.009. 16300771

[ref25] Sul B.; Wallqvist A.; Morris M. J.; Reifman J.; Rakesh V. A computational study of the respiratory airflow characteristics in normal and obstructed human airways. Computers in Biology and Medicine 2014, 52, 130–143. 10.1016/j.compbiomed.2014.06.008. 25058489

[ref26] Martonen T. B.; Guan X. Effects of tumors on inhaled pharmacologic drugs: I. Flow patterns. Cell Biochem Biophys 2001, 35, 233 10.1385/CBB:35:3:233. 11894843

[ref27] Martonen T. B.; Guan X. Effects of Tumors on Inhaled Pharmacologic Drugs: II. Particle Motion. Cell Biochemistry and Biophysics 2001, 35, 245–253. 10.1385/CBB:35:3:245. 11894844

[ref28] Luo H. Y.; Liu Y.; Yang X. L. Particle deposition in obstructed airways. J. Biomech. 2007, 40 (14), 3096–3104. 10.1016/j.jbiomech.2007.03.027. 17499753

[ref29] Srivastav V. K.; Kumar A.; Shukla S. K.; Paul A. R.; Bhatt A. D.; Jain A. Airflow and aerosol-drug delivery in a CT scan based human respiratory tract with tumor using CFD. Journal of Applied Fluid Mechanics 2014, 7 (2), 345–356. 10.36884/JAFM.7.02.20282.

[ref30] Singh D. Numerical assessment of natural respiration and particles deposition in the computed tomography scan airway with a glomus tumour. Proceedings of the Institution of Mechanical Engineers, Part E: Journal of Process Mechanical Engineering 2021, 235 (6), 1945–1956. 10.1177/09544089211024063.

[ref31] Menaissy Y. M; Gal A. A; Mansour K. A Glomus tumor of the trachea. Annals of Thoracic Surgery 2000, 70 (1), 295–297. 10.1016/S0003-4975(00)01285-6. 10921732

[ref32] Fukumitsu K.; Ning Y.; Kanemitsu Y.; Tajiri T.; Okuda K.; Fukuda S.; Uemura T.; Ohkubo H.; Takemura M.; Maeno K.; Ito Y.; Oguri T.; Takakuwa O.; Niimi A. Tracheal Glomus Tumor Complicated with Asthma Exacerbation in a Pregnant Woman. Internal Medicine 2023, 62, 2123–2128. 10.2169/internalmedicine.0510-22. 36450466 PMC10400392

[ref33] Colaut F.; Toniolo L.; Scapinello A.; Pozzobon M. Tracheal glomus tumor successfully resected with rigid bronchoscopy: a case report. Journal of Thoracic Oncology 2008, 3 (9), 1065–1067. 10.1097/JTO.0b013e318183af45. 18758313

[ref34] Daisne J.-F.; Duprez T.; Weynand B.; Lonneux M.; Hamoir M.; Reychler H.; Gregoire V. Tumor volume in pharyngolaryngeal squamous cell carcinoma: comparison at CT, MR imaging, and FDG PET and validation with surgical specimen. Radiology 2004, 233 (1), 93–100. 10.1148/radiol.2331030660. 15317953

[ref35] Chaitanya Thandra K.; Barsouk A.; Saginala K.; Sukumar Aluru J.; Barsouk A. Epidemiology of lung cancer. Contemporary Oncology/Współczesna Onkologia 2021, 25 (1), 45–52. 10.5114/wo.2021.103829. 33911981 PMC8063897

[ref36] Xu G. B.; Yu C. P. Effects of age on deposition of inhaled aerosols in the human lung. Aerosol Sci. Technol. 1986, 5 (3), 349–357. 10.1080/02786828608959099.

[ref37] Hofmann W. Mathematical model for the postnatal growth of the human lung. Respiration Physiology 1982, 49 (1), 115–129. 10.1016/0034-5687(82)90106-2. 7146643

[ref38] Kim J.; Heise R. L.; Reynolds A. M.; Pidaparti R. M. Aging effects on airflow dynamics and lung function in human bronchioles. PLoS ONE 2017, 12 (8), e0183654 10.1371/journal.pone.0183654. 28846719 PMC5573216

[ref39] Lai-Fook S. J.; Hyatt R. E. Effects of age on elastic moduli of human lungs. J. Appl. Physiol. 2000, 89 (1), 163–168. 10.1152/jappl.2000.89.1.163. 10904048

[ref40] Oho K.; Amemiya R. Practical Fiberoptic Bronchoscopy; Igaku-Shoin, 1980.

[ref41] Xi J.; Kim J.; Si X. A.; Corley R. A.; Kabilan S.; Wang S. CFD modeling and image analysis of exhaled aerosols due to a growing bronchial tumor: towards non-invasive diagnosis and treatment of respiratory obstructive diseases. Theranostics 2015, 5 (5), 443 10.7150/thno.11107. 25767612 PMC4350007

[ref42] Menter F. R. Improved two-equation k-omega turbulence models for aerodynamic flows; NASA, 1992.

[ref43] Tiwari A.; Jain A.; Paul A. R.; Saha S. C. Computational evaluation of drug delivery in human respiratory tract under realistic inhalation. Phys. Fluids 2021, 33 (8), 083311 10.1063/5.0053980.

[ref44] Sommerfeld M.; Sgrott O. L. Jr; Taborda M. A.; Koullapis P.; Bauer K.; Kassinos S. Analysis of flow field and turbulence predictions in a lung model applying RANS and implications for particle deposition. European Journal of Pharmaceutical Sciences 2021, 166, 105959 10.1016/j.ejps.2021.105959. 34324962

[ref45] Wedel J.; Steinmann P.; Strakl M.; Hribersek M.; Ravnik J. Can CFD establish a connection to a milder COVID-19 disease in younger people? Aerosol deposition in lungs of different age groups based on Lagrangian particle tracking in turbulent flow. Computational Mechanics 2021, 67 (5), 1497–1513. 10.1007/s00466-021-01988-5. 33758453 PMC7977503

[ref46] Zhang Z.; Kleinstreuer C. Laminar-to-turbulent fluid-nanoparticle dynamics simulations: Model comparisons and nanoparticle-deposition applications. International Journal for Numerical Methods in Biomedical Engineering 2011, 27 (12), 1930–1950. 10.1002/cnm.1447.

[ref47] Islam M. S.; Paul G.; Ong H. X.; Young P. M.; Gu Y. T.; Saha S. C. A review of respiratory anatomical development, air flow characterization and particle deposition. International Journal of Environmental Research Public Health 2020, 17 (2), 380 10.3390/ijerph17020380. 31935991 PMC7014067

[ref48] Rahman M. M.; Zhao M.; Islam M. S.; Dong K.; Saha S. C. Aerosol Particle Transport and Deposition in Upper and Lower Airways of Infant, Child and Adult Human Lungs. Atmosphere 2021, 12 (11), 1402 10.3390/atmos12111402.

[ref49] Islam M. S; Saha S. C; Sauret E.; Ong H.; Young P.; Gu Y. Euler-Lagrange approach to investigate respiratory anatomical shape effects on aerosol particle transport and deposition. Toxicology Research Application 2019, 3, 239784731989467 10.1177/2397847319894675.

[ref50] Rahimi-Gorji M.; Pourmehran O.; Gorji-Bandpy M.; Gorji T.B. CFD simulation of airflow behavior and particle transport and deposition in different breathing conditions through the realistic model of human airways. J. Mol. Liq. 2015, 209, 121–133. 10.1016/j.molliq.2015.05.031.

[ref51] Rahman M. M.; Zhao M.; Islam M. S.; Dong K.; Saha S. C. Aging effects on airflow distribution and micron-particle transport and deposition in a human lung using CFD-DPM approach. Advanced Powder Technology 2021, 32 (10), 3506–3516. 10.1016/j.apt.2021.08.003.

[ref52] Islam M. S.; Gu Y.; Farkas A.; Paul G.; Saha S. C. Helium-oxygen mixture model for particle transport in CT-based upper airways. International journal of environmental research public health 2020, 17 (10), 3574 10.3390/ijerph17103574. 32443715 PMC7277378

[ref53] Farooq U.; Riaz H. H.; Munir A.; Zhao M.; Tariq A.; Islam M. S. Application of heliox for optimized drug delivery through respiratory tract. Phys. Fluids 2023, 35 (10), 103321 10.1063/5.0169934.

[ref54] Zhang W.; Xiang Y.; Lu C.; Ou C.; Deng Q. Numerical modeling of particle deposition in the conducting airways of asthmatic children. Medical Engineering Physics 2020, 76, 40–46. 10.1016/j.medengphy.2019.10.014. 31879223

[ref55] Arsalanloo A.; Abbasalizadeh M.; Khalilian M.; Saniee Y.; Ramezanpour A.; Islam M. S. A computational approach to understand the breathing dynamics and pharmaceutical aerosol transport in a realistic airways. Advanced Powder Technology 2022, 33 (7), 103635 10.1016/j.apt.2022.103635.

[ref56] Kim C. S.; Fisher D. M. Deposition characteristics of aerosol particles in sequentially bifurcating airway models. Aerosol Sci. Technol. 1999, 31 (2–3), 198–220. 10.1080/027868299304255.

[ref57] Feng Y.; Kleinstreuer C. Micron-particle transport, interactions and deposition in triple lung-airway bifurcations using a novel modeling approach. J. Aerosol Sci. 2014, 71, 1–15. 10.1016/j.jaerosci.2014.01.003.

[ref58] Chen X.; Zhong W.; Zhou X.; Jin B.; Sun B. CFD-DEM simulation of particle transport and deposition in pulmonary airway. Powder Technology 2012, 228, 309–318. 10.1016/j.powtec.2012.05.041.

[ref59] Rhodes M. J. Introduction to Particle Technology; John Wiley & Sons, 2008.

[ref60] Watanabe M.; Takagi K.; Ono K.; Aoki T.; Tanaka S.; Shimazaki H.; Aida S.; et al. Successful resection of a glomus tumor arising from the lower trachea: report of a case. Surgery Today 1998, 28 (3), 332–334. 10.1007/s005950050134. 9548322

[ref61] Wu C.; Yan W.; Chen R.; Liu Y.; Li G. Numerical study on targeted delivery of magnetic drug particles in realistic human lung. Powder Technol. 2022, 397, 116984 10.1016/j.powtec.2021.11.028. PMC9110329 35602760

